# Maternal mental health priorities, help-seeking behaviors, and resources in post-conflict settings: a qualitative study in eastern Uganda

**DOI:** 10.1186/s12888-018-1626-x

**Published:** 2018-02-07

**Authors:** Wietse A. Tol, BreeOna Ebrecht, Rebecca Aiyo, Sarah M. Murray, Amanda J. Nguyen, Brandon A. Kohrt, Sheila Ndyanabangi, Stephen Alderman, Seggane Musisi, Juliet Nakku

**Affiliations:** 1 Peter C. Alderman Foundation, plot 855, Mawanda Road, PO Box 20129, Nakawa, Kampala Uganda; 2 Department of Mental Health, Johns Hopkins Bloomberg School of Public Health, 624 N Broadway, Baltimore, MD 21205 USA; 3 University of Virginia Curry School of Education, 405 Emmet St S, Charlottesville, VA 22904 USA; 4 Department of Psychiatry and Behavioral Sciences, George Washington University, 2120 L St NW Suite 600, Washington, DC 20037 USA; 5 Ministry of Health, Republic of Uganda, Plot 6, Lourdel Road, Nakasero, Kampala Uganda; 6 Peter C. Alderman Foundation, New York, NY USA; 7 Department of Psychiatry, Makerere University College of Health Sciences, P.O. Box 7072, Kampala, Uganda; 8 Butabika National Psychiatric Referral Hospital, Butabika Rd, PO Box 7017, Kampala, Uganda

## Abstract

**Background:**

Limited knowledge exists to inform the selection and introduction of locally relevant, feasible, and effective mental health interventions in diverse socio-cultural contexts and health systems. We examined stakeholders’ perspectives on mental health-related priorities, help-seeking behaviors, and existing resources to guide the development of a maternal mental health component for integration into non-specialized care in Soroti, eastern Uganda.

**Methods:**

We employed rapid ethnographic methods (free listing and ranking; semi-structured interviews; key informant interviews and pile sorting) with community health workers (*n* = 24), primary health workers (*n* = 26), perinatal women (*n* = 24), traditional and religious healers (*n* = 10), and mental health specialists (*n* = 9). Interviews were conducted by trained Ateso-speaking interviewers. Two independent teams conducted analyses of interview transcripts following an inductive and thematic approach. Smith’s Salience Index was used for analysis of free listing data.

**Results:**

When asked about common reasons for visiting health clinics, the most salient responses were malaria, general postnatal care, and husbands being absent. Amongst the free listed items that were identified as mental health problems, the three highest ranked concerns were *adeka na aomisio* (sickness of thoughts); *ipum* (epilepsy), and *emalaria* (malaria). The terms epilepsy and malaria were used in ways that reflected both biomedical and cultural concepts of distress. Sickness of thoughts appeared to overlap substantially with major depression as described in international classification, and was perceived to be caused by unsupportive husbands, intimate partner violence, chronic poverty, and physical illnesses. Reported help-seeking for sickness of thoughts included turning to family and community members for support and consultation, followed by traditional or religious healers and health centers if the problem persisted.

**Conclusion:**

Our findings add to existing literature that describes ‘thinking too much’ idioms as cultural concepts of distress with roots in social adversity. In addition to making feasible and effective treatment available, our findings indicate the importance of prevention strategies that address the social determinants of psychological distress for perinatal women in post-conflict low-resource contexts.

**Electronic supplementary material:**

The online version of this article (10.1186/s12888-018-1626-x) contains supplementary material, which is available to authorized users.

## Background

Mental disorders are a leading contributor to ill health in women of reproductive age [1]. The burden of mental ill health in this population is likely higher in low- and middle income countries (LMIC). A recent systematic review and meta-analysis of studies in LMIC found a pooled prevalence of 25.3% for prenatal depression (51 studies from 20 LMIC, *n* = 48,904), and 19.0% for postnatal depression (53 studies from 23 LMIC, *n* = 38,142) [2]. Armed conflicts, which predominantly affect LMIC, are associated with an increased burden of mental ill health. In these settings, women have been found to have higher odds of poor outcomes than men [3].

There is a sizeable body of research on the mental health of populations in both ongoing and post-conflict LMIC settings, but very little of this research focuses specifically on maternal mental health, i.e. the mental health of women during pregnancy or the first post-partum year [4]. Poor maternal mental health, a crucial health concern in its own right, is associated with multiple poor child outcomes including: preterm birth and low birth weight [5]; suboptimal breastfeeding and immunization coverage [6]; being underweight or stunted [7]; higher rates of diarrhea and febrile illness [6, 8]; and, negative impacts on child development [6].

Effective prevention and treatment interventions exist to reduce the burden associated with maternal mental disorders [9, 10], but these are not available to the large majority of women at risk in LMIC. To increase access to care, global mental health researchers and practitioners have called for the integration of evidence-based mental health interventions into non-specialized health systems, such as primary care, maternal and child health care, and community care settings [11, 12]. Answering this call is fraught with multiple challenges. First, relatively little research is available to guide efforts to integrate evidence-based interventions into non-specialized care settings in LMIC. For example, although recent systematic reviews indicate that non-specialized health workers can effectively deliver maternal mental health interventions [13–15], the quality of the evidence is low overall and important gaps remain [15]. Specific gaps in knowledge relate to optimal care delivery models (e.g., collaborative vs. stepped care models), effective training and supervision models for health workers, and the cost-effectiveness of bringing different approaches to scale [15]. Most implementation science research in mental health has been conducted in high-income countries, where uptake of evidence-based interventions in routine clinical settings is also low and lessons learned might not be transferable to LMIC contexts [16, 17].

Second, and more fundamentally, considerable critique has been levied on the field of global mental health for a perceived lack of sensitivity to socio-cultural context. For example, critics have argued that phrasing mental health goals purely in terms of disorders described in psychiatric classification systems from industrialized countries fails to capture locally felt priorities and descriptions of mental health problems in socio-culturally diverse settings [18]. Similarly, viewing intervention options solely in terms of evidence-based mental health services that often originate from research conducted in high-income settings, may undermine existing ways of dealing with common mental disorders [18].

The overall objective of this paper is to describe formative qualitative research for a maternal mental health project in post-conflict Uganda that aimed to address some of these shortcomings in global mental health implementation research. Areas of northern and eastern Uganda experienced an insurgency by the Lord’s Resistance Army between 1987 and 2006 that was associated with widespread human rights violations against the civilian population and the displacement of 1.9 million people at the height of the conflict [19]. The site of this study (the Teso sub-region) also suffered large-scale forced displacement during a 1987–1992 civil war between the Uganda People’s Army and the then newly established government.

Studies in conflict-affected areas in Uganda seven to ten years after cessation of hostilities have found high prevalence of a range of mental health problems, including depression, anxiety, (complex) posttraumatic stress disorder, hazardous alcohol use, as well as suicide [20–22]. Previous qualitative research in Uganda has found that health workers perceive maternal mental health to be a priority, but that government mental health services are generally not available or accessible, prompting the recommendation to make maternal mental health services available at lower levels of the health system [23].

In response to this identified need, a partnership between the Peter C. Alderman Foundation (an international non-governmental organization focused on mental health of violence-affected populations in low-resource areas), the Ugandan Ministry of Health, and Johns Hopkins University was established to develop a mental health services component that can be integrated in the Ugandan maternal and child health care system. Despite challenges in reaching Millennium Development Goals specific to maternal and child health, 95% of pregnant women in Uganda attend at least one antenatal care visit, making antenatal screening an important access point for addressing maternal mental health [24]. Moreover, the government of Uganda is committed to further strengthening maternal and child health services through incorporating the Sustainable Development Goals in its Second National Development Plan [25]. The research described in this paper was implemented as an initial step in designing such a maternal mental health intervention. Our research was framed around two key objectives: (1) given the existence of limited resources and the potential for diverse maternal mental health needs in conflict-affected settings, we aimed to examine the perspectives of stakeholders regarding prioritization of maternal mental health problems; and (2) to build upon local resources and develop an acceptable intervention strategy, we sought to understand current help-seeking patterns for prioritized maternal mental health problems and perceptions of both available resources and gaps in support for women with these problems.

## Methods

### Setting and sampling

**Fig. 1:**
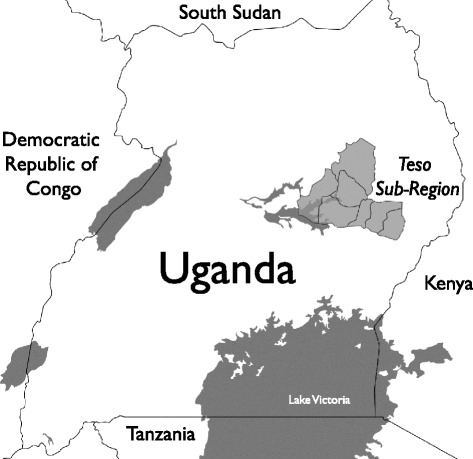
The Teso sub-region study site in eastern Uganda

Research activities were conducted in Soroti district, a predominantly Ateso speaking area of the Teso sub-region of Uganda (Fig. 1, developed by BK) [26]. Uganda’s health care system formally comprises seven levels, corresponding with government administrative divisions. At the village or Health Center I level (HC I), community health workers are organized into Village Health Teams. Village Health Teams are a voluntary health force that generally do not have a physical office and who mainly engage in community-based preventive, health awareness and basic curative activities. At the parish or HC II level, health centers are staffed by primary health care workers (e.g. general nurses or midwives), who deliver basic health services to catchment areas of approximately 5000 people. At the HC III (sub-county level), health centers have a clinical officer, a maternity unit, and unlike lower levels, separate wards for male and female patients (8 beds). Health centers at levels IV-VII have greater staff capacity and provide increasingly specialized care. In Soroti district, there are 14 HC IIs, 12 HC IIIs, two HC IVs, and one 195-bed regional referral hospital.

We estimated that including four of Soroti’s ten sub-counties in our study would give us enough opportunity to identify commonalities and differences between sub-counties and reach data saturation among different participant groups of interest. As the majority of Uganda’s population lives in rural areas where the gap between health needs and available services is greatest, we excluded all sub-counties that did not have a predominantly rural population (*n* = 4) [24]. For feasibility reasons, we then excluded one sub-county where the majority of residents did not speak Ateso. Of the five remaining sub-counties, we randomly selected four for inclusion. Together, these sub-counties represent a predominantly rural, Ateso-speaking population living at various distances from the regional hub in Soroti municipality.

Within each sub-county, we conducted purposive and convenience sampling of stakeholders, including health workers, perinatal women seeking help at health centers, and religious and traditional healers. We aimed to include health workers from different healthcare system levels, with a focus on HCI to HCIII workers as rural women most commonly access antenatal care at these levels. HC IIIs were of particular interest, as these facilities have the highest rates of antenatal care visits. Among sub-counties with multiple HC IIs, HC IIs were randomly selected for study inclusion. In each sub-county, we started recruitment by visiting HC IIs and IIIs during working hours to introduce the study to the clinical officer in-charge of the facility. These clinical officers helped recruit other health workers from their facilities to be interviewed. Community health workers at the HC I level were recruited by liaising with the Village Health Team Sub-County Coordinator.

### Free listing and ranking

An overview of participants and data collection activities can be found in Table 1. We first conducted free listing and ranking (cf. [27, 28]) embedded in semi-structured interviews with 22 primary and 22 community health care workers from HC Is, IIs, and IIIs in each sub-county. Free listing was conducted individually with the exception of one group exercise with community health care workers (Table 1). Specifically, we asked participants *“what are the most common problems for which pregnant women, or women who have just given birth, visit the health center?”* Participants were invited to provide as many answers as possible and were probed to include all of the (physical) health, mental health, social and spiritual or supernatural problems that may lead perinatal women to visit the health center. The interviewer wrote down answers verbatim. Among the problems that were listed, interviewers asked the participants which problems they considered to be related to thoughts, feelings or behaviors (i.e. mental health problems) and to rank the three mental health problems that they felt were most important. Participants were then asked about help-seeking and current health center resources for the three most highly prioritized maternal mental health problems.

**Table 1:** Overview of participants and methods

Research activities	Location	Participants
Free listing and key informant interviews with primary health care workers (AI 1)	Health Center III in SC1, SC2, SC3, SC4 Health Center II in SC1, SC2	*N* = 22 (13 female, 9 male)
Free listing and key informant interviews with community health care workers (AI 2)	Health Center I (Village Health Team) in SC1, SC2	*N* = 22 (7 female, 15 male) – 10 individual interviews and one group interview (12 participants – 8 male and 4 female)
Semi-structured interviews (case vignette) with perinatal women (AI 3)	Health Center III antenatal services in SC1, SC2, SC3, SC4	*N* = 16 perinatal women (4 from each sub-county)
Key informant interviews with primary health care workers (SI 4)	Health Center III in SC2 Health Center II in SC1, SC2	*N* = 4 (female)
Key informant interviews with community health care workers (AI 4)	Health Center I in SC3	*N* = 2 (male)
Key informant interviews with traditional and religious healers (round 1) (AI 5)	SC1, SC2, SC3, SC4	*N* = 8 (4 traditional healers, 4 religious healers; all male)
Key informant interviews with a subset of traditional and religious healers (round 2) (AI 4)	SC1, SC2	*N* = 2 (1 traditional healer, 1 religious healer; both male)
Key informant interviews with perinatal women (AI 6)	SC1, SC2, SC3, SC4	*N* = 8
Key informant interviews with mental health specialists (AI 7)	Health Center V (Soroti Regional Referral Hospital) Health Center VII (Butabika National Referral Hospital, Kampala)	*N* = 9 (3 female, 6 male)

*AI* Additional Information, *SC1* Sub-county 1, as a confidentiality safeguard, sub-counties are numbered

### Semi-structured interviews

We conducted individual semi-structured interviews with perinatal women to gather information on help-seeking behaviors and available services for maternal mental health problems. We aimed to recruit four perinatal women from each included sub-county as they were waiting to receive antenatal care at the HC III. In these interviews, we presented participants with a fictitious case vignette about a woman in Soroti district experiencing mental health problems. The vignette was developed based on descriptions of the most highly prioritized problems identified in the free list interviews with health workers. Participants were asked to describe (1) what the woman in the case vignette could herself do about her problems; (2) their thoughts about where the woman would likely seek help (first, second, third, and so on); (3) to provide details about what services could be obtained at each place where help might be sought; and, (4) what help the health center could offer for this woman and how the health center could improve its assistance.

### Key informant interviews and pile sorting

We conducted key informant interviews with four groups of participants: traditional or religious healers, mental health specialists, community and primary health care workers, and perinatal women. Two traditional or religious healers were recruited through consultation with sub-county leaders and health center officials in each sub-county and nine mental health specialists were recruited from the regional and national referral hospitals. Specialists and healers participated in free listing and ranking exercises during their interviews that were similar in structure to the initial free listing interviews completed with primary and community healthcare workers. In this round of free listing interviews, greater focus was placed on eliciting symptoms of identified priority maternal mental health problems, what groups are particularly affected by these problems, and treatments provided for them.

We then conducted a second round of key informant interviews with a subset of the community and primary health workers who participated in the free listing interviews and traditional healers who participated in the initial key informant interviews. Health workers and healers were selected if they provided rich information about the mental health needs in their sub-counties. We also conducted key informant interviews with two older (more experienced) perinatal women from each sub-county. These women were recruited by health care workers and selected based on their ability to read and their experience with the antenatal care system, as determined by their age and number of previous births. Because free listing resulted in several terms that seemed closely related, we added a pile sorting activity to these key informant interviews. Pile sorting was intended to increase our understanding of how participants would classify various symptoms. Participants were provided with notecards with names of symptoms (in both Ateso and English) derived from descriptions of the three prioritized maternal mental health problems. Participants were asked to sort the cards into piles that made sense for them, in as many or few groups as they preferred, and to name to each pile. These key informant interviews subsequently focused on different idioms of distress used to describe the three prioritized maternal mental health problems; symptoms belonging to these problems; and groups of women particularly affected by them. Finally, participants were asked about the causes of the top three problems and possible solutions for each of the causes. Perinatal women were also probed about help-seeking behaviors for the prioritized problems.

### Instruments, procedures, and ethics

Interviews were conducted using semi-structured interview guides (available as Additional files 1, 2, 3, 4, 5, 6, 7, 8) that contained interview questions, possible probes, and space for notes. They were conducted in Ateso or English, depending on the preferences of the participant. All interviews were audio recorded and transcribed verbatim. When using the guide, interviewers were instructed to still follow the narrative of the participants as much as possible. All interviews were conducted by locally recruited, Ateso-speaking interviewers who participated in a two-week training on research ethics and qualitative interviewing skills. The training provided ample opportunity for skill-based learning through feedback on role-plays, with a strong focus on asking open questions, probing, and giving non-judgmental responses.

All participants provided written informed consent before participating in interviews. Ethical approval for this study was granted by the Johns Hopkins Bloomberg School of Public Health Institutional Review Board, the Mildmay Uganda Research Ethics Committee, and the Uganda National Council for Science and Technology. In addition, we obtained approval from the District Health Officer before starting recruitment.

### Analysis

To analyze free list data and rank priority maternal mental health problems, we used Smith’s Salience Index (S), which ranks responses based on frequency of mention while also weighting items based on where it appeared on participants’ lists. Under this method, frequently mentioned items among individuals are considered to indicate common knowledge or consensus, and items listed earlier are assumed to be more important to the participant. A higher S score is indicative of greater saliency [29]. Smith Salience index is calculated for each item as the total number of items in the list minus the rank order for item *A* divided by the total number of items in the list. Scores range from 0 to approximately 1, with scores closer to 1 indicating greater saliency.

Transcripts were analyzed using an emergent, inductive coding approach [30, 31]. This stage of the analysis was performed in parallel by two teams of analysts: one of postgraduate students based at the Johns Hopkins Bloomberg School of Public Health (JHBSPH) in the US; the other was comprised of one postgraduate student from JHBSPH and a Ugandan community psychology graduate based in Uganda. The US-based team began analysis by conducting an initial transcript review, in which they highlighted and summarized portions of text relevant to the research questions on a sub-set of interviews. Out of these summaries, they developed emergent codes and organized the codes into a framework or codebook. This codebook was utilized to code the interviews using Dedoose software [32]. The Ugandan team manually conducted line-by-line coding for the first three interviews and prepared a list of initial codes. These codes were combined and re-organized into focused codes and the revised codebook consisted of five topical and 41 response codes. New themes emerging from the data were discussed between the researchers and incorporated into the developing codebook as deemed appropriate (available as a Additional file 8). In the last step, the Uganda team coded all interviews using this final codebook, through the aid of the R package for Qualitative Data Analysis software [33].

## Results

**Fig. 2:**
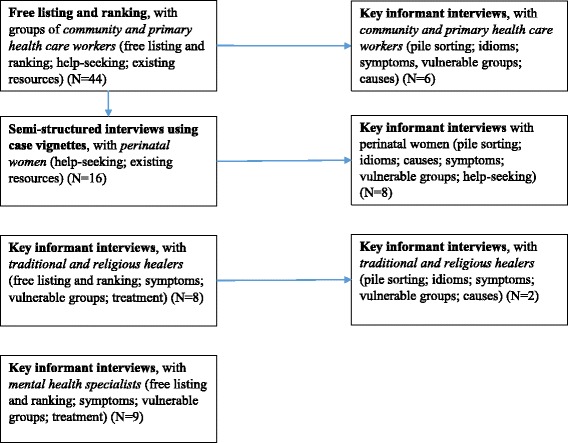
Data collection flow

In total, 81 individual interviews and one 12-participant group interview were conducted. Participants included 51 women and 42 men with an average age of 38 years (Table 1). Of these interviews, 26 were conducted with primary health care workers, 24 with community health care workers, 24 with perinatal women, 10 with traditional healers, and nine with mental health specialists. An overview of the flow of data collection activities is provided in Fig. 2.

### Maternal mental health priorities

The five most salient responses to free lists of common health problems for which women visit the health center were malaria (S = 0.51), general postnatal care (S = 0.32), husbands being absent (S = 0.32); general antenatal check-ups (S = 0.22); and, lower abdominal or stomach pain (S = 0.21). Saliency varied across the different types of participants. For example, “absent husbands” was not one of the five most salient problems listed by primary health care workers, but was the most salient problem for traditional healers. For community health care workers, bleeding and HIV were highly salient, but did not feature in the top five most salient problems for other types of participants.

Of the fifteen problems that were identified as being related to mental health and were listed by two or more respondents (of any type), *adeka na aomisio* (Ateso, literally sickness of thoughts) had the highest saliency (S = 0.28). About half of the participants (20 out of 41) listed *adeka na aomiso* as one of the top three most important mental health problems. The mental health problems with the next highest saliency scores were epilepsy *(ipum)* and malaria *(emalaria)* (Table 2). Although malaria had the highest saliency among the initial free list responses (focused on all health problems), participants ranked it the third most important problem when asked to rank the three most important mental health problems.

**Table 2:** Free listing saliency results for highest priority mental health conditions

Rank	All Respondents	Primary Health Care Workers	Community Health Care Workers	Mental Health Specialists	Traditional Healers
1	Sickness of thoughts (*S* = 0.28)	Sickness of thoughts (S = 0.38)	Epilepsy (S = 0.55)	Postpartum depression and (puerperal) psychosis (S = 0.59)	Absent husbands (S = 0.33)
2	Epilepsy (S = 0.25)	Malaria (S = 0.26)	Malaria (S = 0.37)	Stress (S = 0.26)	Gender based violence (S = 0.29)
3	Malaria (S = 0.20)	Absent husbands (S = 0.23)	Thinking too much and poverty (S = 0.17)	Epilepsy (S = 0.15)	Witchcraft/ spiritual problems (S = 0.29)

S=Smith Salience index

When free list responses from primary health care workers, community health care workers, traditional healers, and mental health specialists were analyzed separately, we found differences in which problems were most salient. Community health care workers listed epilepsy *(ipum)* (S = 0.55) as the most important mental health problem, followed by malaria (S = 0.37), and with *aomom na epol* (thinking too much) and poverty tied for third (S = 0.17). Primary health care workers believed the most important problem was sickness of thoughts (S = 0.38), the second most important was malaria (S = 0.26), and the third most important was absent husbands (S = 0.23). Mental health specialists said the most important problems were postpartum depression and (puerperal) psychosis (S = 0.58), followed by stress (S = 0.26), and epilepsy (S = 0.15). Traditional healers identified absent husbands as the most important mental health problem (S = 0.33), followed by gender-based violence (S = 0.29), and witchcraft or spiritual problems (S = 0.29).

Below we describe the three maternal mental health concerns most commonly prioritized by participants overall. For each concern, we describe participants’ perspectives on common complaints accompanying the concern, causes of the concern, and ways of seeking assistance for managing the concern.

### Sickness of thoughts

Sickness of thoughts *(adeka na aomiso)* was prioritized as the most important mental health problem among perinatal women. Reasons given for prioritizing sickness of thoughts were that it could lead to suicidal or homicidal tendencies and that its causes are highly prevalent in the community. The terms thinking too much *(aomom na epol),* thinking *(aomom)*, over-thinking that leads to ulcers *(akwamakit aomom)* and depression appeared to be used interchangeably by most participants for sickness of thoughts. Perinatal women often referred to thoughts as a tangible force that could be taken away from a woman’s life. They utilized phrases such as – “removing thoughts from the head”, “reduce thinking in the mind”, “remove thoughts from heart”, “make your heart settled”, or “remove burden from life”. Mental health specialists did not utilize the phrase sickness of thoughts, but rather “maternity blues”, which they referred to as distinctly different from postpartum depression.

Symptoms that were listed for sickness of thoughts by primary health care workers and maternity blues by mental health specialists included excessive sadness for longer than two weeks, crying more than usual, low moods, sleep disturbances, less interest in activities that a woman used to enjoy, feeling worthless, outbursts of anger, lack of appetite, weight loss, lack of energy, excessive worries, suicidal thoughts, social withdrawal, fearing life situations, and substance use. Other symptoms mentioned by only a few mental health specialists for maternity blues and only a few primary health care workers for sickness of thoughts were hallucinations, uncoordinated speech, and fever. In contrast, perinatal women and community health care workers were more likely to list behavioral symptoms of sickness of thoughts (sleeping less, crying, not eating and feeling weak) than emotional symptoms (sadness, feelings of worthlessness). One perinatal woman described the symptoms of sickness of thoughts as follows:

The way I can tell that a woman’s life is not okay, she’s growing thin very slowly, she cries about the heart, feels pain in the heart, and also her eating reduces*.*

Perinatal woman, Semi-Structured Interview

Primary health care providers were more likely to give detailed descriptions of symptoms that were consistent with Diagnostic and Statistical Manual fourth version (DSM-IV) criteria, such as stating that the symptoms must be consistently present for two or more weeks. Among both primary health workers and mental health specialists, infanticide (i.e., the drive of a woman to kill her newborn baby), was noted as a very severe symptom of sickness of thoughts or depression.

The most common cause of sickness of thoughts according to participants was absent or unsupportive partners. Perinatal women consistently reported that when husbands in the community provide no support, whether emotional, social or financial, sickness of thoughts was more likely. Because husbands are considered to be responsible for the financial welfare of families, but were perceived to provide very little to no money for the family, food was often reported to be scarce. Women were therefore unable to feed themselves and their growing fetus. One participant explained these burdens:

Mostly here, you may find a mother is depressed… but I think that it’s due to the way they are staying with their husbands in their families. One time a certain lady told me that my husband says I am rude to him, that I am carrying death…. They call pregnancy death… Most women here they get depressed because their husbands, there is a way people here behave. You can find a man and the woman is pregnant; the man goes to the lakeshores for fishing and stays there. You are remaining there, you are alone with the children, feeding becomes a problem, even when you are supposed to go to the hospital but there is nobody to take you, there is no money. Those are the problems they mostly get, you try to refer somebody from here, and she tells you now my husband is not at home and I have children, there is nobody to take care of the children*.*

Enrolled Midwife, Female, Free listing and Ranking Interview

Other common causes given by participants were unwanted or unplanned pregnancies, experiencing domestic violence, being in a “quarreling” relationship, chronic sickness, and poverty.

Healthcare providers also described men refusing to escort their wives to antenatal care visits as a problem, particularly when the men refuse to be tested for HIV. Due to a governmental policy requiring men to be at antenatal care visits, women can be scolded or even turned away from antenatal care if their husbands are not present. Alternatively, if women push their husbands to attend antenatal care, they put themselves at risk for physical violence. Gender-based violence was listed as a very prevalent problem in the community, caused not only by women pushing their husbands to attend antenatal visits, but also by “drunkard” husbands. These men were described as spending most of the day out of the house drinking alcohol and acting violent upon returning home. Health care workers also explained that many women become depressed due to an accumulation of stress in their daily lives related to a combination of intimate partner violence, poverty, HIV, and other diseases that are common but often go untreated. Only mental health specialists mentioned biological causes of depression, such as hormonal imbalances. A Village Health Team member described a common combination of problems as follows:

You find after the mother has got pregnant, you find that people argue, now the woman is pregnant and her mind is not stable. Maybe the husband says that ‘I am not the one responsible for the pregnancy’. So you find her mind is disturbed. It disturbs her, maybe to predict the child, that me, I want a boy… so you find even that disturbs her, “what [gender] child will I produce?” I usually get such women worrying… And another is being attacked. Their husbands, when they come back from drinking they attack you when you are pregnant, you find that woman does not stay well, you find that her body is weak, maybe she is thinking outside that what am I going to do concerning this man’s issues? Whenever he comes from drinking he attacks, whenever he comes you find that her mind is disturbed and not clear. And then after she has delivered, it will find that as he wanted the girl child, she has produced a boy. Then that starts torturing. You will find that such children remain when they have not been taken even for immunization because her mind has not settled to love this baby well*.*

Community Health Care Worker, Male, Focus Group Discussion

Healthcare providers, mental health specialists, and traditional healers described young, single, unemployed, minimally educated women that lack social, financial and partner support as being at the highest risk of developing sickness of thoughts. A religious healer described the reason young women are more affected as:

Most women which are affected, they are young women. These young ladies, you know for them they rush to marry not knowing the challenges which are going to face their families. So they enter in that marriage but the challenges come so they are not able to withstand all that… They are even two in this house here [pointing at a certain house in the neighborhood]. So the man brought one first, left and went and brought another one. This one is still young, one baby only and he has decided to go for another one. That one is also pregnant and the man just took off, he’s not able to answer this problem. So the young ladies are there, they are both pregnant, one is about to give birth*.*

Religious Healer, Male, Key Informant Interview

The most common, and usually first, suggestion made for women who have sickness of thoughts was to pray or talk with someone to “calm their disturbed heart.” One perinatal woman had the following recommendations for a woman affected by sickness of thoughts:

She should find strength. Sometimes she also must go aside and people will give her advice which will give her strength, because when she sits alone she cannot, she needs people to give her some advice which can make her also settle since she’s pregnant. They can make her heart settled so she may stop over thinking. You know when you distance yourself from people you will have many thoughts as it is like that you are even pregnant, the man is treating you badly… now these people need to be near some people who can help you with ideas and others advise*.*

Perinatal Woman, Semi-Structured Interview

Participants said that friends and family could provide encouragement and material or financial support to help a woman experiencing sickness of thoughts. After seeking help from her family and community, participants said the woman should seek out a traditional or spiritual healer. If this healer is unable to help, they should refer the woman to a community health care worker who could help them access the primary health care facility. A primary care nurse described these steps of care-seeking:

What the women do, like some of them who are close to churches can go to churches, they can explain their problems to a pastor or to a spiritual leader who is around then they see how they can counsel her. At times they pray for them also. Then maybe when they see, the church members see that as if the situation is not coming down... They might see that maybe now it is a serious sickness, now, then they refer them to the hospital…then the hospital will now assess and see what the situation is. Like for us here when we assess and we see the situation is not to our handling, we now refer ahead so that that person can be helped…. Others can also walk to their friends. Some can explain to the friends and see how the friends can help them. With counseling maybe some of them can even just leave the home…aaah leave the home and go somewhere else because if the problem is within the home, that’s why you see most women just end up maybe if you are married, somebody leaves that home and maybe goes back to their home where she thinks she can have what…. some peace*.*

Enrolled Nurse, Female, Free Listing and Ranking Interview

Additional resources described at the community level for managing sickness of thoughts included group activities (e.g. village savings and loans or drama groups) and the government or non-governmental organizations that provide food, clothing, advice and protection.

The two health care services for sickness of thoughts described most often were counseling and medication, with medication being utilized most frequently. Primary health care workers described counseling as individual, couple, or family sessions utilized to talk to women about how to deal with their sickness of thoughts. Another reported strategy for managing sickness of thoughts was to treat the underlying diseases or causes of the depression, such as HIV or other chronic illnesses. Some primary health care workers talked about scheduling follow-up appointments or referring patients for care at a higher-level health center. The community health care workers’ role for sickness of thoughts is to sensitize the community about going to the health facility.

### Epilepsy

Epilepsy *(ipum)* was the second most salient mental health problem described by participants. The term epilepsy was used interchangeably with other terms, including falling sickness *(adeka na ebironor),* repeated fits or attacks *(adoenen aria ebironor na ebongonikini)* and fainting *(ailonor).* Though the majority of participants discussed epilepsy as a mental health problem in and of itself, some participants also described feelings of burden among caregivers of a family member or child living with epilepsy. One health care worker described severe consequences of epilepsy:

You will find that people with epilepsy, most of them suffer a lot, most especially when they get the attack. You will find that they suffer a lot. You can find somebody maybe falling on the what? On the fire, some of them have even ended in… recently there is someone who died, he went to fetch water and he fell in a well, killing the person. That’s why I say that number one [epilepsy] should also be dealt with*.*

Enrolled Nurse, Male, Free Listing and Ranking Interview

Symptoms that participants commonly associated with epilepsy included convulsions, falling on the ground, headache, fever, and bruises all over the body. Less commonly, participants listed saliva coming out of the mouth and urinating as symptoms associated with epileptic episodes. Additionally, some perinatal women described that women with epilepsy would have visible wounds on the body due to multiple falls and would be more likely to isolate themselves due to fear of falling in public.

Common causes listed for epilepsy included physical trauma, conflict and war, alcoholism, stress, malaria, fevers, infections of the spinal cord, and family history. One participant explained a perceived link between conflict exposure and the development of epilepsy:

You find it’s common here because the village was attacked by Kony so those bombs and the sound of those guns affected their brains, most of them… So it can be the cause because you will find that here children they have epilepsy but when you maybe ask their background, they cannot even explain it well, that’s why am first querying maybe because the place was first attacked by those people, now that sound of those bombs it can affect their brains*.*

Nursing Assistant, Female, Free Listing and Ranking Interview

Less commonly listed causes included flies, sleeping with someone who has epilepsy, a difficult delivery, and curses or other supernatural causes. Primary health care workers and mental health specialists generally attributed epilepsy to a biological or genetic cause, while community health care workers and perinatal women typically discussed epilepsy as attributable to spiritual or psychosocial factors.

Descriptions of care seeking behaviors for epilepsy were similar to those related to sickness of thoughts. Women were described as first seeking help from their family and friends, followed by a traditional or religious healer. In cases where healers are not able to help women, participants stated they would make health facility referrals. One traditional healer noted two possible treatments for epilepsy:

The solution for the problem [Epilepsy]? As it is the devil, the first solution is to pray a deliverance prayer to the person who is sick or is affected. Another solution: that person can be taken to the hospital for a close checkup*.*

Traditional Healer, Male, Key Informant Interview

Many respondents listed the importance of managing safety concerns for women with epilepsy. They talked of the importance of family and community support, both in reminding a woman to take her medication and in keeping her away from harm.

For them, when somebody has realized they have epilepsy… they should not do risky work like cooking. Like when you are in the kitchen, you should not be alone, you have to be with somebody, when you’re going to the well, make sure you move with somebody. Then there are signs, which happen when maybe if it wants to throw you down, so we always advise them that if you see that sign, you have to come to the hospital… [the families] always help them like fetching water. They always fetch for them water. Like the one I got here, even coming for antenatal they don’t make her come alone. So that when they give them advice on how to feed, what and what, at least that person knows better. The community also always helps them like when a person of epilepsy gets an attack, or falls somewhere and the community is aware that that person has that problem, they will not leave that person. Like if she falls on the road, the community will at least pull that person away from that dangerous place. Because they are aware that she’s having such and such a problem. Though some people have that fear, some of them have that misconception that when you step where that person has fallen you will also automatically get it*.*

Enrolled Nurse, Female, Free Listing and Ranking Interview

Nearly all participants stated that epilepsy should be treated with medication, and that with medication, epilepsy is unlikely to be fatal. However, some health care workers mentioned not knowing the correct medication to give. Respondents also mentioned that risk factors for epilepsy are preventable. The role of health care workers was described as consisting of encouraging mothers with epilepsy to start antenatal care, telling them how to take their medication, and advising them on how to care for themselves. Health care workers also explained that mothers with epilepsy should give birth at facilities via cesarean section.

### Malaria

Participants named both *Emalaria* and *eimiddi,* which translate to malaria, as problems of perinatal women. *Eimiddi* was less frequently listed than *emalaria*. Three women stated that the Ateso phrase *emusuja*, meaning fever, could also be used for malaria. Malaria was listed as a mental health problem by perinatal women, traditional healers, community and primary health care workers, but not by any mental health specialists. Health care workers described malaria as a mental health concern because, with delayed treatment, it was said to cause convulsions or “brain disturbances” and to “spoil the mind of that lady.” Some primary health care workers and mental health specialists referred specifically to cerebral malaria, but most community health care workers and traditional healers referred to it as severe malaria. Aside from cerebral malaria, respondents described malaria as a mental health problem because it can cause stress in mothers.

[In my view] malaria is the second most important problem because most of our mothers suffer from malaria because sometimes we lack drugs and somebody comes here, no treatment, no what, no money so that one also sometimes ends up with stress maybe can even end up with miscarriage*.*

Enrolled Midwife, Female, Free Listing and Ranking Interview

Symptoms described as associated with malaria included: headache, general body weakness, fever, and loss of energy. When perinatal women were asked how they could recognize if someone had malaria, they asserted that infected women would feel warm and might prefer to sit in the sunshine. Participants also mentioned urine discoloration or blood in the urine, especially among pregnant women, and yellow or red discoloration of the eyes, as symptoms of malaria. Two women also reported seeing sores or wounds start to develop in the mouth of someone with malaria.

Nearly all participants reported malaria to be caused by mosquitoes. Less commonly listed causes of malaria were feeling cold, “bad air”, or having the flu. These causes were more likely to be listed by perinatal women or community health care workers.

Health care workers stressed the importance of women coming to the health center when they have malaria. In contrast to sickness of thoughts or epilepsy, participants most commonly said that women with malaria should go directly to health facilities, rather than first going to a family member, friend, or traditional or religious healer. Nearly all participants described the importance of obtaining medication for malaria and taking it as directed by health care workers. The major role that families were seen as being able to take in supporting a woman was to prevent the spread of malaria by making changes to the environment, such as covering holes where standing water collects, shutting windows in the evening to keep mosquitoes out, and clearing bushes from near the house. One community health care worker stated:

*The community needs each family, when the compound is clean, all those broken bottles to be removed from home. You slash the compound also and the home is clean…everyone needs to have a net they sleep under.*

Community Health Care Worker, Male, Free Listing and Ranking Interview

Participants said that health facilities have a responsibility to conduct routine testing for malaria at a woman’s first antenatal visit, provide mosquito nets, and give education on how to prevent malaria. Fansidar, coartem and quinine were the medicines reported to be given to mothers who test positive for malaria. Fansidar was mentioned most frequently as both a front-line treatment and prophylaxis for malaria among pregnant women. In complicated cases or in situations where medications are locally unavailable, participants said pregnant women might be referred to a higher level of care and/or a hospital. The role of community health care workers was described as handing out mosquito nets, providing malaria prevention education, and referring febrile women to health centers. Health workers noted that once women began treatment for malaria, clinics and health teams would provide case management and follow-up to ensure that they completed treatment.

## Discussion

This qualitative study examined stakeholders’ perspectives on maternal mental health priorities, as well as help-seeking and resources for these priorities, in a rural eastern Ugandan post-conflict setting.

Before discussing our findings, we note several study limitations. The study included more formal health actors than actors from outside the formal health system (e.g., traditional and religious healers). This may have led us to uncover biomedical explanatory models in lieu of other understandings of the cause of priority problems. Nonetheless, the main mental health concern we identified, sickness of thoughts, is not a recognized medical diagnosis. Further, malaria, which is not traditionally seen as a mental health concern by medical specialists, was highly prioritized. These findings suggest that we were successful in identifying concerns outside of biomedical perspectives. We did include perinatal women and a sizeable group of community health workers who have no training in mental health, and thus arguably are more likely to articulate lay perspectives than medical views on mental health problems. Second, perinatal women were recruited during antenatal visits and interviews were held at the health centers. This may have led to the inclusion of perinatal women with a more favorable opinion towards health centers or encouraged women to give responses perceived as desirable by health workers. Third, we employed a rapid ethnographic approach, involving locally trained interviewers from the same socio-cultural background as the participants. A hesitancy to explain concepts in detail to an interviewer who was perceived to share the same cultural knowledge may have limited our ability to understand complex social phenomena. Due to a lack of multiple follow-up interviews with participants, our process may also have produced a more superficial understanding of concepts than a longer-term ethnographic study. Further, only one Ugandan was involved in data analysis.

Our study does have several key strengths, including triangulation across different data collection techniques and types of participants, while also having sufficient breadth and depth of information to explore priorities and reach saturation in our analyses within each participant group. Analyses were conducted by two independent teams of analysts, strengthening the trustworthiness of the coding procedures.

Pertaining to our first research objective, we found *adeka na aomisio* (or sickness of thoughts) to be the most commonly prioritized maternal mental health concern. Although there was some variation across types of participants (e.g., traditional healers appeared to prioritize determinants of psychological distress rather than a specific psychological condition), there was broad consensus about the importance of sickness of thoughts. Sickness of thoughts was largely used interchangeably with thinking too much, an idiom of distress that has been identified in 138 studies across diverse socio-cultural contexts [34] and is listed as a cultural concept of distress in the DSM-5 [35]. In this study, psychological problems reported as associated with sickness of thoughts by non-health workers were primarily observable signs rather than symptoms of depression (e.g., eating less, crying, and sleep disturbance).

Social scientists have warned against conflating thinking too much idioms with psychiatric disorder categories, as idioms of distress can communicate important social dimensions of suffering and often do not exist as orderly syndromes [34]. Our findings indeed seem to indicate that the sickness of thoughts idiom conveys a constellation of social adversity experienced by pregnant and postpartum women in Soroti. This constellation includes high levels of intimate partner violence, unsupportive spouses (including for reproductive health) who potentially drink alcohol, financial stress, and physical illnesses (e.g. HIV/AIDS and malaria). Concerns on social origins of maternal distress expressed by traditional and religious healers overlapped with those expressed by health workers, specifically the perceived influence of unsupportive spouses and intimate partner violence. While epidemiological research in eastern Uganda has found that prior conflict experiences predicted higher levels of intimate partner violence [36], we note that this constellation of adversity for pregnant and postpartum women has been found to underlie maternal common mental disorders in low-resource settings more broadly [37–40]. More in-depth ethnographic and social epidemiological research would help elucidate the complex socio-cultural dynamics that underlie sickness of thoughts, including its etiology in the historical context of armed conflict and ongoing adversity.

*Ipum* (epilepsy), also referred to by participants as falling sickness (*adeka na ebironor*), repeated fits or attacks (*adoenen aria ebironor na ebongonikini*), and fainting (*ailonor*), was prioritized as the second most important perinatal mental health concern. Epilepsy is not a highly prevalent disorder in eastern Africa; a meta-analysis estimated its prevalence as 5.1 per 1000 people, though given the stigma associated with epilepsy, this may be an underestimation [41]. However, compared to other regions, epilepsy is about twice as common in sub-Saharan Africa [41], and an analysis of primary care health information system data found that it is the most common presenting mental, neurological, or substance use related complaint in refugee settings [42]. Given that participants in this study indicated that conflict, stress, and evil spirits may cause *ipum*, its high prioritization may be in part due to an overlap with non-epileptic fits in the context of spirit possession. Previous research in southern Uganda has found that dissociative complaints, which are linked to the experience of potentially traumatic events, may include fits [43, 44].

Similarly, the term malaria may be syncretic, reflecting both biomedical and cultural concepts of distress. In neighboring Kenya, Jenkins and colleagues have noted that common mental disorders are often misdiagnosed as malaria in primary care [45], and Ndetei and colleagues included reporting malaria in a screener for common mental disorders [46]. Likewise, Tesfaye and colleagues have hypothesized that malaria is often misdiagnosed in people with psychological symptoms visiting primary care facilities in Ethiopia [47].

Consistent with studies in other settings, we found that participants emphasized prayer and social support from family and community members (including emotional encouragement and financial support) to address *adeka na aomisio* [34]. Assistance from a traditional healer, religious healer, or community health worker was said to be sought in cases where sickness of thoughts persists. Descriptions of help-seeking pathways for epilepsy were similar, except that spiritual support was said to specifically address evil spirits as a cause of epilepsy, safety concerns were emphasized, and medication was a commonly discussed treatment option. For malaria, women were described as seeking help directly from health centers.

Based on these qualitative findings and international guidelines for managing perinatal depression, a treatment strategy can be developed that has local relevance (i.e., is focused on locally prioritized mental health concerns) and is feasible (i.e., considers existing help-seeking behaviors and resources). For example, the UK National Institute for Health and Clinical Excellence (NICE) guidelines for perinatal mental health emphasize a stepped care approach in which women, identified in primary care settings, receive progressively more specialized treatments depending on need [48]. In this context, identification of perinatal mental health problems in primary care should focus on sickness of thoughts. Because of the overlap of sickness of thoughts with perinatal depression, cultural adaptation of existing screening measures for major depressive episodes seems a feasible next step [49]. Identification of sickness of thoughts should take place in an accessible health care setting (e.g. routine antenatal care), given that help-seeking for sickness of thoughts only occurs at health centers at later stages.

In the process of intervention design, we feel it would be important to consult with local stakeholders to collaboratively design specific proposed intervention components. A promising public health strategy would be to address the identified constellation of social adversity as a determinant of sickness of thoughts through a preventive approach [50]. For example, research in urban Uganda found a community mobilization program effective in primary and secondary prevention of intimate partner violence [51]. A gender-informed psycho-educational intervention for both mothers and fathers focused on the spousal relationship and parenting in Australian primary care was found to have preventive benefits for perinatal mental health [52]. An important direction for future implementation science would be to assess whether socio-culturally adapted prevention interventions with demonstrated effectiveness in other settings [9, 53] can be successfully delivered in low-resource post-conflict contexts [17].

## Conclusion

In conclusion, our qualitative study identified sickness of thoughts, epilepsy, and malaria to be the highest prioritized maternal mental health concerns in a post-conflict eastern Ugandan setting. Sickness of thoughts was perceived to result from a combination of social adversity including intimate partner violence, unsupportive spouses, alcohol misuse, poverty, and physical illnesses. In addition to integrating feasible and effective treatment strategies into existing maternal and child health care, a fruitful direction for mental health interventions concerns preventive interventions that address the social determinants of sickness of thoughts. Malaria and epilepsy deserve further study as syncretic idioms that incorporate both biomedical and cultural concepts of distress.

## Additional files

Additional file 1: Key Informant Interviews. Primary Health Care Workers (HC-III). (DOCX 308 kb)

Additional file 2: Group Interviews. Community Health Workers and Primary Health Care Workers. (DOCX 325 kb)

Additional file 3: Semi-structured Interviews. (DOCX 305 kb)

Additional file 4: Key Informant Interviews. Service Providers. (DOCX 322 kb)

Additional file 5: Key Informant Interviews. Traditional and Religious Healers. (DOCX 309 kb)

Additional file 6: Key Informant Interviews. Perinatal Women. (DOCX 328 kb)

Additional file 7: Key Informant Interviews. Mental Health Specialists. (DOCX 309 kb)

Additional file 8: CODEBOOK (Combined from Uganda and US-based teams). (DOCX 24 kb)

## Abbreviations

DSM-IV: Diagnostic and Statistical Manual of Mental Disorders - fourth version
HC: Health Center
JHBSPH: Johns Hopkins Bloomberg School of Public Health
LMIC: Low- and Middle- Income Countries
NICE: UK National Institute for Health and Clinical Excellence
S: Smith’s Salience Index
