# Supplementary material for: Maternal mental health priorities, help-seeking behaviors, and resources in post-conflict settings: a qualitative study in eastern Uganda
Source: BMC Psychiatry. 2018 Feb 7;18:39. doi: 10.1186/s12888-018-1626-x (PMC5803865; doi:10.1186/s12888-018-1626-x)
Supplement: Supplementary file 1 — Key Informant Interviews. Primary Health Care Workers (HC-III). (DOCX 308 kb) [file 12888_2018_1626_MOESM1_ESM.docx]

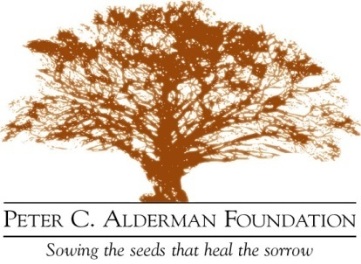

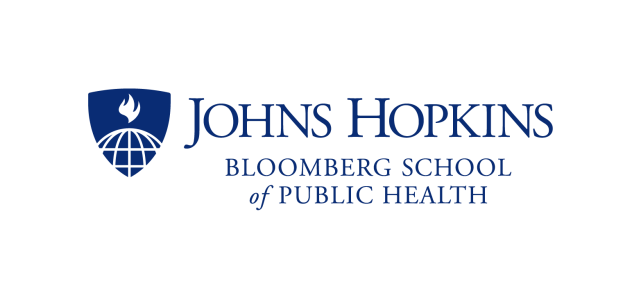

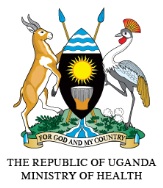


**Key Informant Interviews**

**Primary Health Care Workers (HC-III)**

**Overview of procedures**

| 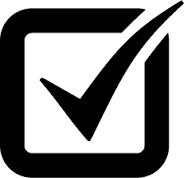 |  |
| --- | --- |
| 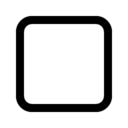 | **STEP 1. Informed consent (on a separate form)**  In this part we ask the key informant if they would like to be interviewed or not. |
| 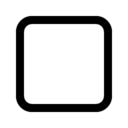 | **STEP 2. The interview**  If the key informant agrees to be interviewed, we give more information about the interview in this part. We also fill out the information required on this page. |
| 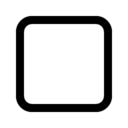 | **STEP 3. The interview**  In this part we introduce a number of themes and ask the key informant for their response. Remember: in a semi-structured interview, you do not have the follow the themes in the order on paper. You can be flexible, depending on how the key informant prefers to give their responses. You can follow their choice of order, as long as you cover all the themes. |
| 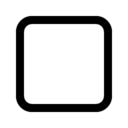 | **STEP 4. Closing**  In this part we thank the key informant for their contributions, and emphasize again the confidentiality of the information. |

| Participant code: __________________________  Participant name: __________________________  Participant address: __________________________  Participant phone #: __________________________  Date: __________________________  Location of interview: __________________________  Time interview started: __________________________  Time interview finished: __________________________  Interviewer: __________________________  **THIS PAPER IS KEPT SEPARATE FROM THE FOLLOWING PAPERS WITH INTERVIEW DATA** |
| --- |

**STEP 2. INTRODUCTION**

**[READ OUT LOUD]**

Thank you very much for being willing to be interviewed. As I said earlier, we would like to discuss the topic of maternal mental health with you. With maternal mental health we mean the health of women who are pregnant, or who have recently given birth. With mental health problems we mean problems related to thinking, feeling, or behavior. Your opinions will be helpful to develop an action plan for how to deal with maternal mental health problems in Soroti.

We would like to record the conversation so we do not miss any important information. This is the tape recorder [**SHOW THE RECORDER**], which I will put here.

Do you have any questions for us?

**STEP 3. INTERVIEW**

Before we start, can I ask a few short questions first?

| Gender (as observed): __________________________  Age: __________________________  Place of work: __________________________  Position: __________________________  Length of time in that position: __________________________ |
| --- |

| **THEME 1** | **[READ OUT LOUD]**  In the Group Interviews, you and your colleagues discussed the three most important mental health problems that women may have. I would like to ask your further expert opinion about these problems.  For each of these problems, could you give me a more detailed description?  Let’s start with [**NAME MOST IMPORTANT PROBLEM FROM GROUP INTERVIEW]**  [**ASK PROBES AND FILL OUT IN TABLE**] |
| --- | --- |

| **[COPY “1. MOST IMPORTANT PROBLEM FROM GROUP INTERVIEW” HERE]** | |
| --- | --- |
| Symptoms**:**  How would one recognize that a woman has [**NAME OF PROBLEM**]? |  |
| Affected groups:  Which groups of women are particularly affected by **[NAME OF PROBLEM]?** |  |

| **[COPY “2. SECOND MOST IMPORTANT PROBLEM FROM GROUP INTERVIEW” HERE]** | |
| --- | --- |
| Symptoms**:**  How would one recognize that a woman has [**NAME OF PROBLEM**]? |  |
| Affected groups:  Which groups of women are particularly affected by **[NAME OF PROBLEM]?** |  |

| **[COPY “3. THIRD MOST IMPORTANT PROBLEM FROM GROUP INTERVIEW” HERE]** | |
| --- | --- |
| Symptoms**:**  How would one recognize that a woman has [**NAME OF PROBLEM**]? |  |
| Affected groups:  Which groups of women are particularly affected by **[NAME OF PROBLEM]?** |  |

| **THEME 2** | What do you think causes these problems?  [**FIRST LIST ALL THE CAUSES THAT THE PARTICIPANT CAN THINK OF. THEN, MOVE TO THE NEXT QUESTION**] |
| --- | --- |

| **THEME 3** | Can anything be done to address these *causes* of the problems that you have mentioned? |
| --- | --- |

| **[COPY “1. MOST IMPORTANT PROBLEM FROM GROUP INTERVIEW” HERE]** | **Cause 1:** | **Solution:** |
| --- | --- | --- |
|  | **Cause 2:** | **Solution:** |
|  | **Cause 3:** | **Solution:** |
|  | **Cause 4:** | **Solution:** |
|  | **Cause 5:** | **Solution:** |
| **[COPY “2. SECOND MOST IMPORTANT PROBLEM FROM GROUP INTERVIEW” HERE]** | **Cause 1:** | **Solution:** |
|  | **Cause 2:** | **Solution:** |
|  | **Cause 3:** | **Solution:** |
|  | **Cause 4:** | **Solution:** |
|  | **Cause 5:** | **Solution:** |
| **[COPY “3. THIRD MOST IMPORTANT PROBLEM FROM GROUP INTERVIEW” HERE]** | **Cause 1:** | **Solution:** |
|  | **Cause 2:** | **Solution:** |
|  | **Cause 3:** | **Solution:** |
|  | **Cause 4:** | **Solution:** |
|  | **Cause 5:** | **Solution:** |

| **THEME 4** | What do you personally currently do when you meet women with these problems?  Which of these activities do you think helps most to deal with the problem? |
| --- | --- |

| **[COPY “1. MOST IMPORTANT PROBLEM FROM GROUP INTERVIEW” HERE]** | |
| --- | --- |
| **Activity** | **How helpful? (1. Not helpful; 2. A little bit helpful; 3. Very helpful)** |
|  |  |
|  |  |
|  |  |
|  |  |
|  |  |

| **[COPY “2. SECOND MOST IMPORTANT PROBLEM FROM GROUP INTERVIEW” HERE]** | |
| --- | --- |
| **Activity** | **How helpful? (1. Not helpful; 2. A little bit helpful; 3. Very helpful)** |
|  |  |
|  |  |
|  |  |
|  |  |
|  |  |

| **[COPY “3. THIRD MOST IMPORTANT PROBLEM FROM GROUP INTERVIEW” HERE]** | |
| --- | --- |
| **Activity** | **How helpful? (1. Not helpful; 2. A little bit helpful; 3. Very helpful)** |
|  |  |
|  |  |
|  |  |
|  |  |
|  |  |

**STEP 4. CLOSING**

[**READ OUT LOUD**]

Thank you very much for your help!

As I said before, we will not share your information with others. We will keep the recording and notes in a secure place. We will keep your name separately from the recording and the notes, and we will keep your name also in a secure place.

Any questions before we finish?

Thank you very much again.
