# Supplementary material for: Maternal mental health priorities, help-seeking behaviors, and resources in post-conflict settings: a qualitative study in eastern Uganda
Source: BMC Psychiatry. 2018 Feb 7;18:39. doi: 10.1186/s12888-018-1626-x (PMC5803865; doi:10.1186/s12888-018-1626-x)
Supplement: Supplementary file 2 — Group Interviews. Community Health Workers and Primary Health Care Workers. (DOCX 325 kb) [file 12888_2018_1626_MOESM2_ESM.docx]

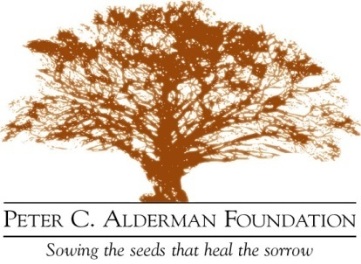

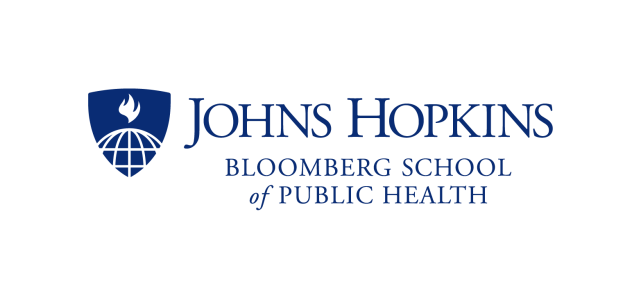

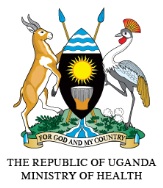


**Group Interviews**

**Community Health Workers and Primary Health Care Workers**

**Overview of procedures**

| 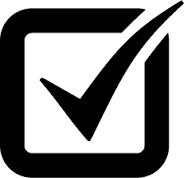 |  |
| --- | --- |
|  | **STEP 1. Recruitment (with group of health workers – see recruitment script)**  In this part we introduce our research activities to the health workers |
| 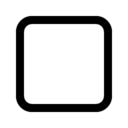 | **STEP 2. Informed consent (individually – on a separate form)**  In this part we ask permission to ask questions in a group discussion. |
| 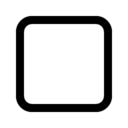 | **STEP 3. Introduction**  In this part we set the ground rules for the discussion. |
| 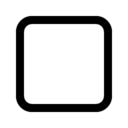 | **STEP 4. Registration form**  In this part we ask participants to state their gender, position, and how long they have worked in that position. We also fill out the information on this page. |
| 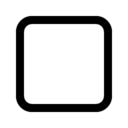 | **STEP 5. Discussion**  In this part we ask the group a number of questions, in a fixed format. |
| 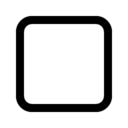 | **STEP 6. Closing**  In this part we thank the participants for their contributions, and emphasize again the confidentiality of the information. |

| [**FILL OUT**]  Date: __________________________  Location of interview: __________________________  Time interview started: __________________________  Time interview finished: __________________________  Facilitator: __________________________  Note taker: __________________________ |
| --- |

**STEP 2. INTRODUCTION**

**[READ OUT LOUD]**

Thank you very much for being willing to join this group interview. As we said earlier, we would like to ask your opinions about maternal mental health in Soroti. With maternal mental health we mean the mental health of women who are pregnant, or who have recently given birth. With mental health problems we mean problems related to thinking, feeling, or behavior. Your opinions will be helpful to develop an action plan for how to deal with maternal mental health problems in Soroti.

My name is **[NAME OF THE FACILITATOR]**, and I will lead the discussion. My colleague’s name is **[NAME OF THE NOTE-TAKER]**, and s/he will take notes. We would like to record the conversation so we do not miss any important information. This is the tape recorder [**SHOW THE RECORDER**], which I will put in the middle [**TURN ON RECORDER**].

Before we start, I would like to ask if you could fill your gender, your position in the Health Center, and the amount of years you have worked in the Health Center.

**[HAND OUT THE REGISTRATION LIST]**

In the next 1 hour to 1.5 hours, I would like to do the following:

1. I introduce a question and ask you to discuss this. Me, or the note taker, may ask questions in between your discussion
2. For the note taker it would really help if one person speaks at a time
3. I would like to encourage everyone in the group to participate in answering the questions
4. We are asking about the experiences of people in your community, not your own experiences. If you would like to discuss your own experiences with us, we are more than happy to do this after the group interview.
5. There are no right or wrong answers. We are interested in your opinion.

Please remember, I would like us to focus on women who are pregnant, or who have just given birth.

Before we start, do you have any questions for us?

**STEP 3. REGISTRATION FORM**

| **Number** | **1. Male**  **2. Female** | **Position** | **Years in position** |
| --- | --- | --- | --- |
| 1 |  |  |  |
| 2 |  |  |  |
| 3 |  |  |  |
| 4 |  |  |  |
| 5 |  |  |  |
| 6 |  |  |  |
| 7 |  |  |  |
| 8 |  |  |  |
| 9 |  |  |  |
| 10 |  |  |  |
| 11 |  |  |  |
| 12 |  |  |  |

**Numbers based on the FGD sitting arrangement figure:**

**RECORDER**

**Facilitator**

**Note-taker**

**STEP 4. DISCUSSION**

| **THEME 1** | **What are the most common problems for which pregnant women, or women who have just given birth, visit this health center? Please list as many problems as you can think of.**  **[FILL OUT THE ANSWERS IN THE TABLE BELOW]**  **[ENCOURAGE BY SAYING] What *other* reasons do pregnant women, or women who have just given birth, have to visit this health center?**  **[FINISH UNTIL THE GROUP CANNOT LIST ANY MORE PROBLEMS]**  **[THEN, ASK FOR A SHORT DESCRIPTION OF EACH PROBLEM]**  **[MAKE A NOTE *BY YOURSELF* IF THE PROBLEM IS A MENTAL HEALTH PROBLEM OR NOT]** |
| --- | --- |
| Probes: | - What kind of health problems do pregnant/ postnatal women have? - What kind of mental health problems do pregnant/ postnatal women have?   For example, problems with *feelings* (such as sadness or worries), *thoughts* (such as trouble concentrating, thinking strange things), *behaviors* (such as doing things that are out of the ordinary, )   - What kind of social problems do pregnant/ postnatal women have? - What kind of spiritual/ supernatural problems do pregnant/postnatal women have? |

| **Problem** | **Short description** | **Mental health problem? [CIRCLE]** |
| --- | --- | --- |
|  |  | Yes  No |
|  |  | Yes  No |
|  |  | Yes  No |
|  |  | Yes  No |
|  |  | Yes  No |
|  |  | Yes  No |
|  |  | Yes  No |
|  |  | Yes  No |
|  |  | Yes  No |
|  |  | Yes  No |
|  |  | Yes  No |

| **I would like to focus on mental health problems specifically. That is, problems related to feelings, thoughts, and behaviors. You mentioned:**  **[READ OUT LOUD THE *MENTAL HEALTH* PROBLEMS FROM THE TABLE. THEN, ASK]**  **Which are the three most important problems that pregnant/ postnatal women may have according to the group?** |
| --- |

| **1. First most important problem:** | **Why is this the most important problem?** |
| --- | --- |
|  |  |
| **2. Second most important problem** | **Why is this the second most important problem?** |
|  |  |
| **3. Third most important problem** | **Why is this the third most important problem?** |
|  |  |

| **THEME 2** | **[READ OUT LOUD]**  **Thank you. That is very helpful.**  **Now, I would like to ask you what people do to deal with the 3 most important maternal mental health problems you mention. For each problem, I would like to ask:**  **What do *women* do for themselves to deal with this problem?**  **What do *families* do to deal with this problem?**  **What does the *community* do to deal with this problem?**  **What does the *health center* do to deal with this problem?**  **Let’s start with the most important problem.** |
| --- | --- |

| [**WRITE DOWN “1. MOST IMPORTANT PROBLEM” HERE:**] |
| --- |
| **What do women do for themselves to deal with [NAME MOST IMPORTANT PROBLEM]?** |
|  |
|  |
|  |
|  |
|  |
| **What do women’s families do to deal with [NAME MOST IMPORTANT PROBLEM]?** |
|  |
|  |
|  |
|  |
|  |
| **What does the community do to deal with [NAME MOST IMPORTANT PROBLEM]?** |
|  |
|  |
|  |
|  |
|  |

| **What does the health center do to deal with [NAME MOST IMPORTANT PROBLEM]?** |
| --- |
|  |
|  |
|  |
|  |
|  |

| [**WRITE DOWN “2. SECOND MOST IMPORTANT PROBLEM HERE”**] |
| --- |
| **What do women do for themselves to deal with [NAME SECOND MOST IMPORTANT PROBLEM]?** |
|  |
|  |
|  |
|  |
|  |
| **What do women’s families do to deal with [NAME SECOND MOST IMPORTANT PROBLEM]?** |
|  |
|  |
|  |
|  |
|  |
| **What does the community do to deal with [NAME SECOND IMPORTANT PROBLEM]?** |
|  |
|  |
|  |
|  |
|  |

| **What does the health center do to deal with [NAME SECOND MOST IMPORTANT PROBLEM]?** |
| --- |
|  |
|  |
|  |
|  |
|  |

| [**WRITE DOWN “3. THIRD MOST IMPORTANT PROBLEM HERE”**] |
| --- |
| **What do women do for themselves to deal with [NAME THIRD MOST IMPORTANT PROBLEM]?** |
|  |
|  |
|  |
|  |
|  |
| **What do women’s families do to deal with [NAME THIRD MOST IMPORTANT PROBLEM]?** |
|  |
|  |
|  |
|  |
|  |
| **What does the community do to deal with [NAME THIRD MOST IMPORTANT PROBLEM]?** |
|  |
|  |
|  |
|  |
|  |

| **What does the health center do to deal with [NAME THIRD MOST IMPORTANT PROBLEM]?** |
| --- |
|  |
|  |
|  |
|  |
|  |

| **THEME 3** | **[READ OUT LOUD]**  **Thank you. That is very helpful.**  **Finally, I would like to ask your opinions on what can be done better at your health center to deal with these problems. In other words, what can different people in your health center do to improve the problems that you have mentioned?**  **Let’s start with the most important problem.** |
| --- | --- |

| [**WRITE DOWN “1. MOST IMPORTANT PROBLEM” HERE:**] | |
| --- | --- |
| **Person**  (for example, Village Health Team member, midwife, nurse, clinical officer) | **What can this person do to improve [NAME MOST IMPORTANT PROBLEM]** |
|  |  |
|  |  |
|  |  |
|  |  |
|  |  |
|  |  |
|  |  |

| [**WRITE DOWN “2. SECOND MOST IMPORTANT PROBLEM” HERE:**] | |
| --- | --- |
| **Person**  (for example, Village Health Team member, midwife, nurse, clinical officer) | **What can this person do to improve [NAME SECOND MOST IMPORTANT PROBLEM]?** |
|  |  |
|  |  |
|  |  |
|  |  |
|  |  |
|  |  |
|  |  |

| [**WRITE DOWN “3. THIRD MOST IMPORTANT PROBLEM” HERE:**] | |
| --- | --- |
| **Person**  (for example, Village Health Team member, midwife, nurse, clinical officer) | **What can this person do to improve [NAME THIRD MOST IMPORTANT PROBLEM]?** |
|  |  |
|  |  |
|  |  |
|  |  |
|  |  |
|  |  |
|  |  |

**STEP 5. CLOSING**

[**READ OUT LOUD**]

Thank you very much for your help!

As I said before, we are asking everyone not to share the information with others. For ourselves, we will keep the recording and notes in a secure place. We will keep your names separately from the recording and the notes, and we will keep your names also in a secure place.

We are asking the same questions with other Health Centers. Once we have summarized everybody’s opinions, we will be back. We will provide a summary of the answers to you. Then, we would like to ask your opinion on a plan of action that we will develop based on your opinions and suggestions.

Any questions before we finish?

Thank you very much again.
