# Supplementary material for: Maternal mental health priorities, help-seeking behaviors, and resources in post-conflict settings: a qualitative study in eastern Uganda
Source: BMC Psychiatry. 2018 Feb 7;18:39. doi: 10.1186/s12888-018-1626-x (PMC5803865; doi:10.1186/s12888-018-1626-x)
Supplement: Supplementary file 3 — Semi-structured Interviews. (DOCX 305 kb) [file 12888_2018_1626_MOESM3_ESM.docx]

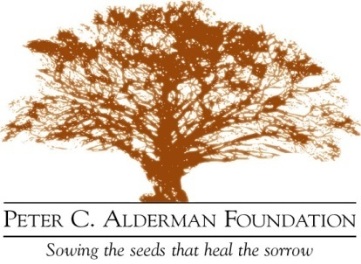

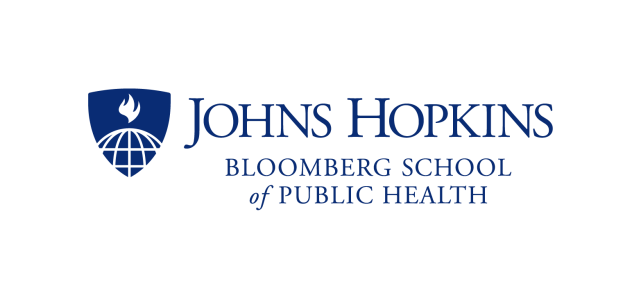

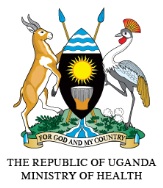


**Semi-structured Interviews**

**Perinatal women seeking assistance in Health Center**

**Overview of procedures**

***STEP 1. Informed consent (on a separate form)***

In this part we ask the woman visiting the Health Center if they would like to be interviewed or not.

***STEP 2. The interview***

If the woman visiting the Health Center agrees to be interviewed, we give more information about the interview in this part.

***STEP 3. The interview***

In this part we introduce a made-up example of a woman experiencing psychological difficulties. Thinking about this example, we ask the woman visiting the Health Center questions. Remember: in a semi-structured interview, you do not have the follow the themes in the order on paper. You can be flexible, depending on how the interview participant prefers to answer. You can follow their choice of order, as long as you cover all the themes.

***STEP 4. Closing***

In this part we thank the woman visiting the Health Center for their contributions, and emphasize again the confidentiality of the information.

**STEP 2. INTRODUCTION**

**[READ OUT LOUD]**

Thank you very much for being willing to be interviewed. As I said earlier, we would like to discuss the topic of women’s mental health in Soroti with you. Your opinions will be helpful to develop an action plan for how to deal with mental health problems in women in Soroti.

We would like to record the conversation so we do not miss any important information. This is the tape recorder [**SHOW THE RECORDER**], which I will put here.

In the next 1 hour, I would like to do the following:

1. I will read out the example of a woman experiencing some difficulties. Let us call her Angelica. Angelica is not a real person. But, we made up her story after listening to the real experiences of women in Soroti district.
2. Keeping the made up example of Angelica in mind, I would like to ask you some questions. These questions are about what kind of things could help Angelica with her difficulties. You are free to share your personal experiences if you have had similar experiences. But this is not necessary. We are asking about how women in your community overall would respond if a woman had problems like Angelica.

Before we start, do you have any questions for us?

**STEP 3. DISCUSSION**

| **Case Vignette** | **[READ THE FOLLOWING OUT LOUD]**  **I am going to tell a story about a women named Angelica. Angelica is experiencing difficulties in her life. As I said before, Angelica is not a real woman. We made her up. But, we made up her story after listening to the experiences of real women in Soroti district. Please listen closely to her story. After that I will ask you some questions. These questions are about what people like Angelica could do about their difficulties.**  ***Angelica was born in a town 20 kilometers from Soroti Town. She was born in the time of the Teso Rebellion (1985). Her parents were farmers, and had five children besides Angelica. Her father had two wives. For some time her parents lived in Katakwi camp. Angelica was growing up happily and married in 2003. In the year she was married the LRA came to Soroti. Angelica’s parents’ house was attacked during the nighttime, and she lost her father and brother. Her husband was abducted by the LRA and she later heard he was killed in an ambush.***  ***After some years of very hard life in Soroti town, Angelica found a new husband. She moved with her husband to a smaller town in Soroti district. Her husband often stays out late and comes home drunk, and then beats Angelica. He also treats her badly in other ways. Angelica has had three children from this marriage and one from her previous husband. The family is having trouble finding enough money for school and clothes for the children and themselves. Right now, she is pregnant of the fourth child from her husband.***  ***Angelica often thinks about her difficulties. She has headaches and feels weak, and she does not sleep well. This happens especially in periods when she is very worried about her family. There are periods when Angelica finds it very difficult to get out of bed and talk to people in her neighborhood. In these periods she is not very patient with her children. She also has periods in which she has nightmares about what happened on the night the LRA came to her village.***  ***Now that Angelica is pregnant again, she has been more worried than usual. She has been sleeping very little and is thinking too much. She does not feel like eating food. She does not speak much with other people and has pains in her body. She is afraid of what will happen in the future and often feels her heart pounding loudly. Sometimes she thinks things would be better if she were dead. She has been crying a lot when she is by herself.*** |
| --- | --- |

| **THEME 1** | **[READ OUT LOUD]**  **Thank you for listening.**  **What do you think Angelica could do for herself about her difficulties? What could she do on her own that would make life easier for her?** |
| --- | --- |

| 1. |
| --- |
| 2. |
| 3. |
| 4. |
| 5. |
| 6. |
| 7. |
| 8. |
| 9. |
| 10. |

| **THEME 2** | **[READ OUT LOUD]**  **Thank you. That is very helpful.**  **Maybe Angelica could also look for help from others. Where do you think Angelica could go to get help with her problems? Where could she go first, and where could she go second, and third? What kind of help could she get at that place?** |
| --- | --- |

| **1. First place to seek help?** | **What kind of help could she get at that place?** |
| --- | --- |
|  |  |
| **2. Second place to seek help?** | **What kind of help could she get at that place?** |
|  |  |
| **3. Third place to seek help** | **What kind of help could she get at that place?** |
|  |  |
| **4. Fourth place to seek help** | **What kind of help could she get at that place?** |
|  |  |
| **5. Fifth place to seek help** | **What kind of help could she get at that place?** |
|  |  |

| **THEME 3** | **[IF HEALTH CENTER WAS NOT MENTIOND BY PARTICIPANTS]**  **[READ OUT LOUD]**  **What do you think could be done for Angelica in the *health center*? For which problems could that be helpful?** |
| --- | --- |

| Helping activity in health center | Problems for which this would be helpful |
| --- | --- |
|  |  |
|  |  |
|  |  |
|  |  |
|  |  |
|  |  |
|  |  |
|  |  |
|  |  |
|  |  |

| **THEME 4** | **[READ OUT LOUD]**  **Thank you. That is very helpful.**  **Finally, I would like to ask your opinions on what can be done better at your health center to assist Angelica. In other words, what can different people in your health center do to improve the problems that Angelica has?** |
| --- | --- |

| **Person**  (for example, Village Health Team member, midwife, nurse, clinical officer) | **What can this person do?** |
| --- | --- |
|  |  |
|  |  |
|  |  |
|  |  |
|  |  |
|  |  |
|  |  |

**STEP 4. CLOSING**

[**READ OUT LOUD**]

Thank you very much for your help!

As I said before, we will not share your information with others. We will keep the recording and notes in a secure place. We will keep your name separately from the recording and the notes, and we will keep your name also in a secure place.

Any questions before we finish?

Thank you very much again.
