# Supplementary material for: Maternal mental health priorities, help-seeking behaviors, and resources in post-conflict settings: a qualitative study in eastern Uganda
Source: BMC Psychiatry. 2018 Feb 7;18:39. doi: 10.1186/s12888-018-1626-x (PMC5803865; doi:10.1186/s12888-018-1626-x)
Supplement: Supplementary file 4 — Key Informant Interviews. Service Providers. (DOCX 322 kb) [file 12888_2018_1626_MOESM4_ESM.docx]

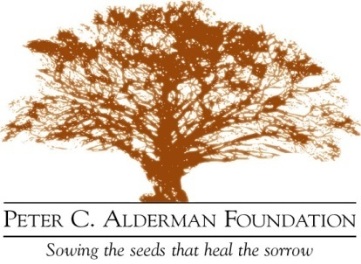

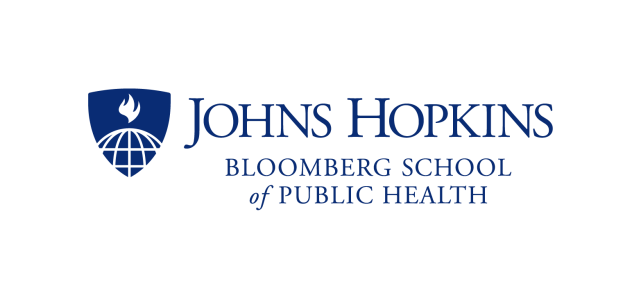

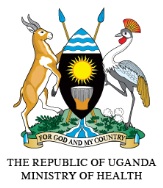


**Key Informant Interviews**

**Service Providers**

**Overview of procedures**

| 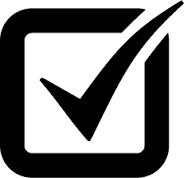 |  |
| --- | --- |
| 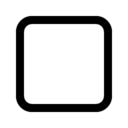 | **STEP 1. Informed consent (on a separate form)**  In this part we ask the key informant if they would like to be interviewed or not. |
| 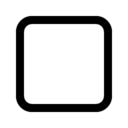 | **STEP 2. The interview**  If the key informant agrees to be interviewed, we give more information about the interview in this part. We also fill out the information required on this page. |
| 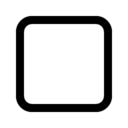 | **STEP 3. The interview**  In this part we introduce a number of themes and ask the key informant for their response. Remember: in a semi-structured interview, you do not have the follow the themes in the order on paper. You can be flexible, depending on how the key informant prefers to give their responses. You can follow their choice of order, as long as you cover all the themes. |
| 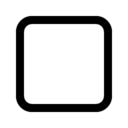 | **STEP 4. Closing**  In this part we thank the key informant for their contributions, and emphasize again the confidentiality of the information. |

| Participant code: __________________________  Participant name: __________________________  Participant address: __________________________  Participant phone #: __________________________  Date: __________________________  Location of interview: __________________________  Time interview started: __________________________  Time interview finished: __________________________  Interviewer: __________________________  **THIS PAPER IS KEPT SEPARATE FROM THE FOLLOWING PAPERS WITH INTERVIEW DATA** |
| --- |

**STEP 2. INTRODUCTION**

**[READ OUT LOUD]**

Thank you very much for being willing to be interviewed. As I said earlier, we would like to discuss the topic of maternal mental health with you. With maternal mental health we mean the health of women who are pregnant, or who have recently given birth. With mental health problems we mean problems related to thinking, feeling, or behavior. Your opinions will be helpful to develop an action plan for how to deal with maternal mental health problems in Soroti.

We would like to record the conversation so we do not miss any important information. This is the tape recorder [**SHOW THE RECORDER**], which I will put here.

Do you have any questions for us?

**STEP 3. INTERVIEW**

Before we start, can I ask a few short questions first?

| Gender (as observed): __________________________  Age: __________________________  Place of work: __________________________  Position: __________________________  Length of time in that position: __________________________ |
| --- |

| **THEME 1** | **[READ OUT LOUD]**  **We are first going to begin with an exercise. I have written the Ateso names for different difficulties that people may have on these cards. I’d like you to sort them into groups that make sense to you. You can make as many or as few groups as you like.**  **[Fill out the table below by writing in the symptoms they sorted into each group]**  **[Ask about problems they were hesitant about or wanted to include in two group]** |
| --- | --- |
| Probes: | - Is there a name for all of the difficulties that fall into group 1? How about group 2? group 3? - Can you think of any other symptoms or difficulties that could fall into pile 1? Pile 2? Etc. |

|  | **Group 1**  **[Name of Problem]** | **Group 2**  **[Name of Problem]** | **Group 3**  **[Name of Problem]** | **Group 4**  **[Name of Problem]** | **Group 5**  **[Name of Problem]** |
| --- | --- | --- | --- | --- | --- |
| 1 |  |  |  |  |  |
| 2 |  |  |  |  |  |
| 3 |  |  |  |  |  |
| 4 |  |  |  |  |  |
| 5 |  |  |  |  |  |
| 6 |  |  |  |  |  |
| 7 |  |  |  |  |  |
| 8 |  |  |  |  |  |
| 9 |  |  |  |  |  |
| 10 |  |  |  |  |  |

***NOTE: If the respondent did not list a term similar to Depression, Malaria or Epilepsy please skip Theme 2 and continue to Theme 3.**

| **THEME 2** | **[READ OUT LOUD]**  **Thank you for participating in this activity. We are going to use the groups you have created to answer the rest of the questions in this interview. First, we would like to understand some of the other common terms for your group(s).** |
| --- | --- |
| Probes: | - If they named one of their groups something similar to “depression” or “thinking too much” ask if their term is the same or different as the following: **Adeka na aomiso, aomom na epol, aomom, akwamakit aomom.** Have them explain any differences. - If they named one of their groups something similar to “Epilepsy”, “Fainting” or “Falling”, ask if it is the same or different as the following: **Ikwinykwiny, adeka na ailonor/eilonor, ailonor/eilonor, ipum, adeka na ecakanari, adeka na adoenen.** Have them explain any differences. - If they named one of their groups something similar to “Malaria” or “Fever”, ask if it is the same or different as the following terms: **Emusuja, eimidi, emalaria** Have them explain any differences. |

| **If participant grouped something similar to Depression or Thinking too much:**  **[NAME OF GROUP]:_______________________________** | |
| --- | --- |
| - Adeka na aomiso | Differences: |
| - Aomom na epol, | Differences: |
| - Aomom | Differences: |
| - Akwamakit aomom | Differences: |
| If participant grouped something similar to Epilepsy, Fainting or Falling:  [NAME OF GROUP]:_______________________________ | |
| - Ikwinykwiny | Differences: |
| - Ailonor/eilonor | Differences: |
| - Adeka na ailonor/eilonor | Differences: |
| - Ipum | Differences: |
| - Adeka na ecakanari | Differences: |
| - Adeka na adoenen | Differences: |
| If participant grouped something similar to Malaria or Fever:  [NAME OF GROUP]:_______________________________ | |
| - Emusuja | Differences: |
| - Eimidi | Differences: |
| - Emalaria | Differences: |

| **THEME 3** | **[READ OUT LOUD]**  Thank you, that is very helpful. Now, I would like to ask you for some more details about these problems.  Let’s start with [**NAME OF GROUP 1]** |
| --- | --- |

| **[COPY THE NAME OF “Group 1” HERE]** | |
| --- | --- |
| Symptoms**:**  How would one recognize that a woman has [**NAME OF PROBLEM**]? |  |
| Affected groups:  Which groups of women are particularly affected by **[NAME OF PROBLEM]?** |  |

| **[COPY THE NAME OF “Group 2” HERE]** | |
| --- | --- |
| Symptoms**:**  How would one recognize that a woman has [**NAME OF PROBLEM**]? |  |
| Affected groups:  Which groups of women are particularly affected by **[NAME OF PROBLEM]?** |  |

| **[COPY THE NAME OF “Group 3” HERE]** | |
| --- | --- |
| Symptoms**:**  How would one recognize that a woman has [**NAME OF PROBLEM**]? |  |
| Affected groups:  Which groups of women are particularly affected by **[NAME OF PROBLEM]?** |  |

| **[COPY THE NAME OF “Group 4” HERE]** | |
| --- | --- |
| Symptoms**:**  How would one recognize that a woman has [**NAME OF PROBLEM**]? |  |
| Affected groups:  Which groups of women are particularly affected by **[NAME OF PROBLEM]?** |  |

| **[COPY THE NAME OF “Group 5” HERE** | |
| --- | --- |
| Symptoms**:**  How would one recognize that a woman has [**NAME OF PROBLEM**]? |  |
| Affected groups:  Which groups of women are particularly affected by **[NAME OF PROBLEM]?** |  |

| **THEME 4** | Thank you. Now we are going to talk about what you think causes these problems.  What do you think causes **[NAME OF “Group 1]**?  [**FIRST LIST ALL THE CAUSES THAT THE PARTICIPANT CAN THINK OF. THEN, MOVE TO THE NEXT QUESTION**] |
| --- | --- |

| **THEME 5** | Can anything be done to address these *causes* of the problems that you have mentioned? |
| --- | --- |

| **[COPY NAME OF “GROUP 1” HERE]** | **Cause 1:** | **Solution:** |
| --- | --- | --- |
|  | **Cause 2:** | **Solution:** |
|  | **Cause 3:** | **Solution:** |
|  | **Cause 4:** | **Solution:** |
|  | **Cause 5:** | **Solution:** |
| **[COPY NAME OF “Group 2” HERE]** | **Cause 1:** | **Solution:** |
|  | **Cause 2:** | **Solution:** |
|  | **Cause 3:** | **Solution:** |
|  | **Cause 4:** | **Solution:** |
|  | **Cause 5:** | **Solution:** |
| **[COPY NAME OF “Group 3” HERE]** | **Cause 1:** | **Solution:** |
|  | **Cause 2:** | **Solution:** |
|  | **Cause 3:** | **Solution:** |
|  | **Cause 4:** | **Solution:** |
|  | **Cause 5:** | **Solution:** |
| **[COPY NAME OF “Group 4” HERE]** | **Cause 1:** | **Solution:** |
|  | **Cause 2:** | **Solution:** |
|  | **Cause 3:** | **Solution:** |
|  | **Cause 4:** | **Solution:** |
|  | **Cause 5:** | **Solution:** |
| **[COPY NAME OF “Group 5” HERE]** | **Cause 1:** | **Solution:** |
|  | **Cause 2:** | **Solution:** |
|  | **Cause 3:** | **Solution:** |
|  | **Cause 4:** | **Solution:** |
|  | **Cause 5:** | **Solution:** |

| **THEME 6** | What do you personally do when you meet women with these problems?  Which of these activities do you think helps most to deal with the problem? |
| --- | --- |

| **[COPY NAME OF “Group 1” HERE]** | |
| --- | --- |
| **Activity** | **How helpful? (1. Not helpful; 2. A little bit helpful; 3. Very helpful)** |
|  |  |
|  |  |
|  |  |
|  |  |
|  |  |

| **[COPY NAME OF “Group 2” HERE]** | |
| --- | --- |
| **Activity** | **How helpful? (1. Not helpful; 2. A little bit helpful; 3. Very helpful)** |
|  |  |
|  |  |
|  |  |
|  |  |
|  |  |

| **[COPY NAME OF “Group 3” HERE]** | |
| --- | --- |
| **Activity** | **How helpful? (1. Not helpful; 2. A little bit helpful; 3. Very helpful)** |
|  |  |
|  |  |
|  |  |
|  |  |
|  |  |

| **[COPY NAME OF “Group 4” HERE]** | |
| --- | --- |
| **Activity** | **How helpful? (1. Not helpful; 2. A little bit helpful; 3. Very helpful)** |
|  |  |
|  |  |
|  |  |
|  |  |
|  |  |

| **[COPY NAME OF “Group 5” HERE]** | |
| --- | --- |
| **Activity** | **How helpful? (1. Not helpful; 2. A little bit helpful; 3. Very helpful)** |
|  |  |
|  |  |
|  |  |
|  |  |
|  |  |

**STEP 4. CLOSING**

[**READ OUT LOUD**]

Thank you very much for your help!

As I said before, we will not share your information with others. We will keep the recording and notes in a secure place. We will keep your name separately from the recording and the notes, and we will keep your name also in a secure place.

Any questions before we finish?

Thank you very much again.
