# Supplementary material for: Maternal mental health priorities, help-seeking behaviors, and resources in post-conflict settings: a qualitative study in eastern Uganda
Source: BMC Psychiatry. 2018 Feb 7;18:39. doi: 10.1186/s12888-018-1626-x (PMC5803865; doi:10.1186/s12888-018-1626-x)
Supplement: Supplementary file 5 — Key Informant Interviews. Traditional and Religious Healers. (DOCX 309 kb) [file 12888_2018_1626_MOESM5_ESM.docx]

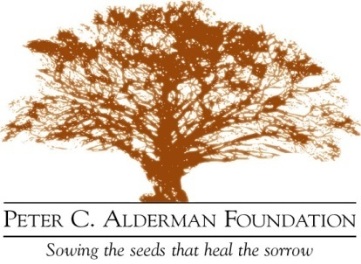

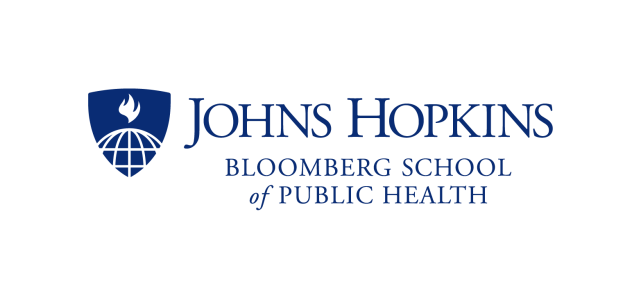

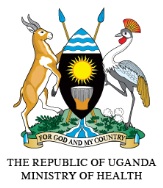


**Key Informant Interviews**

**Traditional and Religious Healers**

**Overview of procedures**

| 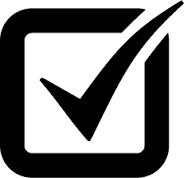 |  |
| --- | --- |
| 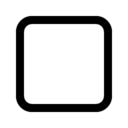 | **STEP 1. Informed consent (on a separate form)**  In this part we ask the key informant if they would like to be interviewed or not. |
| 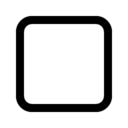 | **STEP 2. Introduction**  If the key informant agrees to be interviewed, we give more information about the interview in this part. We also fill out the information required on this page. |
| 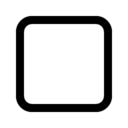 | **STEP 3. The interview**  In this part we introduce a number of themes and ask the key informant for their response. Remember: in a semi-structured interview, you do not have the follow the themes in the order on paper. You can be flexible, depending on how the key informant prefers to give their responses. You can follow their choice of order, as long as you cover all the themes. |
| 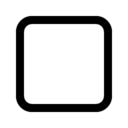 | **STEP 4. Closing**  In this part we thank the key informant for their contributions, and emphasize again the confidentiality of the information. |

| Participant code: __________________________  Date: __________________________  Location of interview: __________________________  Time interview started: __________________________  Time interview finished: __________________________  Interviewer: __________________________ |
| --- |

**STEP 2. INTRODUCTION**

**[READ OUT LOUD]**

Thank you very much for being willing to be interviewed. As I said earlier, we would like to discuss the topic of maternal mental health with you. With maternal mental health we mean the mental health of women who are pregnant, or who have recently given birth. Your opinions will be helpful to develop an action plan for how to deal with maternal mental health problems in Soroti.

We would like to record the conversation so we do not miss any important information. This is the tape recorder [**SHOW THE RECORDER**], which I will put here.

Do you have any questions for us?

**STEP 3. INTERVIEW**

Before we start, can I ask a few short questions first?

| Gender (as observed): __________________________  Age: __________________________  Place of work: __________________________  Position: __________________________  Length of time in that position: __________________________ |
| --- |

| **THEME 1** | **What are the common reasons why women visit you for help? Please tell us as many problems as you can think of. Please remember, we are focused on women who are pregnant, or women who have just given birth.**  **[FILL OUT THE ANSWERS IN THE TABLE BELOW]**  **[ENCOURAGE BY SAYING] What *other* reasons do these women have to visit you for help?**  **[FINISH UNTIL THE KEY INFORMANT CANNOT LIST ANY MORE PROBLEMS]**  **[THEN, ASK FOR A SHORT DESCRIPTION OF EACH PROBLEM]**  **[MAKE A NOTE *BY YOURSELF* IF THE PROBLEM IS A MENTAL HEALTH PROBLEM OR NOT]** |
| --- | --- |
| Probes: | - What kind of health problems do pregnant/postnatal women have? - What kind of mental health problems do pregnant/postnatal women have?   For example, problems with *feelings* (such as sadness or worries), *thoughts* (such as trouble concentrating, thinking strange things), *behaviors* (such as doing things that are out of the ordinary, )   - What kind of social problems do pregnant/postnatal women have? - What kind of spiritual/ supernatural problems do pregnant/postnatal women have? |

| **Problem** | **Short description** | **Mental health problem? [CIRCLE]** |
| --- | --- | --- |
|  |  | Yes  No |
|  |  | Yes  No |
|  |  | Yes  No |
|  |  | Yes  No |
|  |  | Yes  No |
|  |  | Yes  No |
|  |  | Yes  No |
|  |  | Yes  No |
|  |  | Yes  No |
|  |  | Yes  No |
|  |  | Yes  No |
|  |  | Yes  No |

| **I would like to focus on mental health problems specifically. That is, problems related to feelings, thoughts, and behaviors. You mentioned:**  **[READ OUT LOUD THE *MENTAL HEALTH* PROBLEMS FROM THE TABLE. THEN, ASK]**  **Of these problems, which are the three most important problems for pregnant women, or women who have just given birth, in your opinion?** |
| --- |

| **1. First most important problem:** | **Why is this the most important problem?** |
| --- | --- |
|  |  |
| **2. Second most important problem** | **Why is this the second most important problem?** |
|  |  |
| **3. Third most important problem** | **Why is this the third most important problem?** |
|  |  |

| **THEME 2** | **[READ OUT LOUD]**  Thank you, that is very interesting. I would like to ask some more information about the three most important problems for women who are pregnant or who have just given birth that you mentioned.  For each of these problems, could you give me a more detailed description?  Let’s start with [**NAME MOST IMPORTANT PROBLEMS FROM THEME 1]**  [**ASK PROBES AND FILL OUT IN TABLE**] |
| --- | --- |

| **[COPY “1. MOST IMPORTANT PROBLEM FROM THEME 1” HERE]** | |
| --- | --- |
| Symptoms**:**  How would one recognize that a woman has [**NAME OF PROBLEM**]? |  |
| Affected groups:  Which groups of women are particularly affected by **[NAME OF PROBLEM]?** |  |

| **[COPY “2. SECOND MOST IMPORTANT PROBLEM FROM THEME 1” HERE]** | |
| --- | --- |
| Symptoms**:**  How would one recognize that a woman has [**NAME OF PROBLEM**]? |  |
| Affected groups:  Which groups of women are particularly affected by **[NAME OF PROBLEM]?** |  |

| **[COPY “3. THIRD MOST IMPORTANT PROBLEM FROM THEME 1” HERE]** | | |
| --- | --- | --- |
| Symptoms**:**  How would one recognize that a woman has [**NAME OF PROBLEM**]? | |  |
| Affected groups:  Which groups of women are particularly affected by **[NAME OF PROBLEM]?** | |  |
| **THEME 3** | **What do you do to help women who have these problems? Can you explain exactly how you do this treatment from beginning to end?** | |
|  | Probes:  - What do you call this treatment?  - How do you know this treatment will solve this problem?  - How do you give this treatment? What are the different steps involved?  - What happens after the treatment? | |

| **[COPY “1. MOST IMPORTANT PROBLEM FROM THEME 1” HERE]** | |
| --- | --- |
| Name of treatment |  |
| Treatment selection |  |
| Treatment steps |  |
| After the treatment |  |

| **[COPY “2. SECOND MOST IMPORTANT PROBLEM FROM THEME 1” HERE]** | |
| --- | --- |
| Name of treatment |  |
| Treatment selection |  |
| Treatment steps |  |
| After the treatment |  |

| **[COPY “3. THIRD MOST IMPORTANT PROBLEM FROM THEME 1” HERE]** | |
| --- | --- |
| Name of treatment |  |
| Treatment selection |  |
| Treatment steps |  |
| After the treatment |  |

**STEP 4. CLOSING**

[**READ OUT LOUD**]

Thank you very much for your help!

As I said before, we will not share your information with others. We will keep the recording and notes in a secure place. We will keep your name separately from the recording and the notes, and we will keep your name also in a secure place.

Any questions before we finish?

Thank you very much again.
