# Supplementary material for: Maternal mental health priorities, help-seeking behaviors, and resources in post-conflict settings: a qualitative study in eastern Uganda
Source: BMC Psychiatry. 2018 Feb 7;18:39. doi: 10.1186/s12888-018-1626-x (PMC5803865; doi:10.1186/s12888-018-1626-x)
Supplement: Supplementary file 8 — CODEBOOK (Combined from Uganda and US-based teams). (DOCX 24 kb) [file 12888_2018_1626_MOESM8_ESM.docx]

**CODEBOOK (Combined from Uganda and US-based teams)**

| **No.** | **Mnemonic or**  **numeric “brief”**  **code** | **Full description of code** | **When to use and when not use the code. Examples of use of the code to assist coders.** |
| --- | --- | --- | --- |
|  | **Most important problems** | identifies what the respondent says is the most important problems | Use to code problems respondent identifies as one of three most important |
| 1.0 | **Clinic visits** | describes general health problems and/or reasons for clinic visits | Note that this and subcodes are coded “clinic visits” because the question asks what kind of problems women come to the clinic before. But can be used more generally to code health problems women have, regardless of whether they come to clinic for these problems. Use subcodes when applicable. (Exception: for clinic visits - mental health, this refers only to clinic visits. mental health problems should be coded separately using mental health codes) |
| 1.1 | Clinic visits_mother physical | Physical problems of the mother that she may initially present to the clinic for – not mental health specific | Use this to code for general physical problems **and** reasons women come to clinic (eg: pain, malaria, epilepsy, utis, etc.). Do not use for mental health symptoms or postnatal problems. Use code 1.4 for that. |
| 1.2 | Clinic visits_child physical | Physical problems of the child that she may initially present to the clinic for – not mental health specific | Use this to code for both general physical problems **and** reasons women might bring their child to the clinic (eg:, illness, injury). Do not use for mental health symptoms |
| 1.3 | Clinic visits_routine | Preventative care, family planning, etc. regular clinical visit and check-ups | Use for clinic visits related to general primary health care, family planning, prevention...not related to illness. Can be visit for either mother or child (eg: immunizations) |
| 1.4 | Clinic visits_perinatal | Physical problems women face post-partum | Use for health issues and/or clinic visits for issues like bleeding, blood loss, infection, sepsis, pre-eclampsia |
| 1.5 | Clinic visits_ mental health problems | Issues related to decline or poor mental wellbeing | Use this code for clinic visits related to mental health problems. To describe mental health problems more generally, use “mental health” codes and subcodes |
| 1.6 | Clinic visits_headaches | Headaches | Use code for issues concerning headaches. |
| 3.1 | HIV+ mother | Issues related to being an HIV+ woman not related to child transmission | Use this code to describe general issues related to HIV (eg: opportunistic infections, treatment adherence). Do not use for issues specific to having child with HIV or preventing transmission to child |
| 3.2 | HIV+ mother_PMTCT | Treatment adherence and early intervention for children of HIV+ mothers after giving birth | Use for issues such as infant care, medication related to prevention of HIV transmission to child |
|  | Stressors_general | Describes any type of psychosocial stressor experienced by the mother not included in the codes below | Use to code comments or issues the respondent reports as being stressful (i.e. not coder judgement). For example, problems in the family (not related to the spouse - for that use codes in Domestic Problems below) |
|  | Stressors_conflict-related | Used to describe stressors related to war, political instability | eg: bomb blasts, child soldiers, etc. |
| 2.0 | Stressors_parenting | This code describes issues related to child or parenting that the respondent identifies as a stressor | Use this code for general parenting problems related to children, for example, birth defects, child accidents, too many children. Do not use for birth/parenting stressors related to HIV. Do not use for general physical child health problems that present at clinic (eg: child fever) |
| 4.0 | Stressors_Perinatal | Used to describe pregnancy stress that doesn’t fall under any other subcategory | Use if the code is related to pregnancy or post-natal period and stress but not specific to pregnancy status. (eg: Things like no rest, no recovery time, etc.) |
| 4.1 | Stressors_Pregnancy | Used to describe stressors related specifically to pregnancy | Use for issues specifically related to pregnancy status, not other psychosocial stressors within the perinatal period (examples for use:unexpected pregnancy, unwanted pregnancy, abortion contemplation, pregnancy due to rape, too many children, poorly timed,). Do not use for infertility. |
|  | Stressors_infertility | Used to describe stressors related specifically to problems getting / staying pregnant | Eg: miscarriages, can’t get pregnant |
| 7.1 | Substance use | Describes any substance use or problems related to substance use | Use for references to alcohol, drug, cigarette, or other use or problems associated with the use of substances |
| 8.0 | Mental problem | General description of mental problems/mental distress not otherwise specified | Use only if not included in below specifiers or if respondent doesn’t get specific |
| 8.1 | Mental problem_Depression | Issues related to depression. | Use for statements made about or exclusively stated as depression. According to the respondent. |
| 8.2 | Mental problem_Bereavement | Issues related to the period of grief or mourning after the loss of a loved one | Use for references to loss, grief or mourning of a loved or close one |
| 8.3 | Mental problem_Suicide | Issues related to suicide attempt, suicidal ideation/contemplation | Use for references made to woman wanting to kill herself or attempting to do so. |
| 8.4 | Mental problem_Thinking | Issues related to overthinking, thinking too much, intrusive thoughts, negative thinking | Use for statements made about women over thinking or having too many thoughts that interfere with the quality of life |
| 8.5 | Mental problem_Anxiety | Issues related to anxiety | Use for references made to anxiety or feelings of anxiousness. |
| 8.7 | Mental problem_Psychosis/mania | Describes any abnormal or psychotic behavior seen in the woman | Use in reference to abnormal behaviors exhibited by woman according to respondent. Distinguish from mental decline related to malaria and epilepsy. Examples: mad-woman, run mad |
| 8.8 | Mental problem_stress | Describes mental problems discussed as stress | Use this rather than the general mental problem code if the respondent describes stress |
| 8.9 | Mental problem_trauma | Describes mental health problems related to a traumatic event | use to describe symptoms or problems experienced following/in relation to a traumatic event, violence |
| 9.0 | Spiritual issues | Related to spiritual issues, evil spirits, ghosts, other problems | Use code for references made to eccelatestrial issues that specifically cause a problem or concern for the woman. Do not use in reference to using spirituality as a means for support. |
| 9.1 | spiritual issues_witchcraft | subcategory of spiritual issues related to witchcraft | use to describe problems/actions related to witchcraft, distinct from evil spirits |
| 9.2 | spiritual issues_evil spirits | subcategory of spiritual issues related to witchcraft | use to describe problems/actions related to evil spirits, distinct from witchcraft |
| 11.0 | Domestic problems | General description of domestic problems experienced between a couple | Use only if not included in below specifiers or if respondent doesn’t get specific |
| 11.1 | Domestic problems_infidelity | Describes cheating/polygamy and associated problems. | Use code for statements regarding a cheating partner (eg: concubine, unfaithful, stepping out etc.) |
| 11.2 | Domestic problems_violence | Describes intimate partner violence, either physical or sexual. | Use for descriptions of physical or sexual violence. Do not use for descriptions of intimate partner sexual problems that are not described as involving violence. For example, man wanting to have sex too soon after birth would not be coded here unless it was specified that sex was forced. Instead, for something like that it could be coded as “domestic problem” and double coded as “perinatal stressor”, |
| 11.3 | Domestic problems_neglect | Lack of support from partner – often referred to as “neglect” in interviews | Use for descriptions of abandonment, lack of support from partner. Does not include polygamy/cheating, which would fall under infidelity. Examples include descriptions of failing to fulfill obligations, failing to provide, or “neglect”. |
| 12.0 | Malaria | A general code for references related to malaria. | Use for issues related to malaria. Can be double-coded with other categories (support, treatment, etc.). Do not use for descriptions of brain deterioration or mental problems due to malaria. Instead use 12.1 Malaria_mental for that. |
| 12.1 | Malaria_mental | Describes brain deterioration, psychosis, or other mental health problems attributed to malaria | Use when respondent describes mental health problems due to malaria. Do not use if malaria is discussed more generally but not in the context or description of a mental problem. |
| 13.0 | Epilepsy | Use to describe issues related to epilepsy. | Use this code to describe problems/symptoms associated with epilepsy. Examples would be “epileptics faint, have seizures”. If symptoms commonly associated with epilepsy are described but the respondent attributes these to psychosis rather than epilepsy, use that code instead. |
|  | Low Education | Describes lack of basic knowledge related to health care, child care, etc. | Eg: mother is ignorant, mother does not know about services, etc. |
|  | Nutrition | Anything related to nutrition of mother, family, child | Example: mother does not want to eat, has a poor diet. Do not use for supports - use support_diet/hygiene instead |
| 4.0 | Hygiene | Anything related to water, sanitation, hygiene | Example: mother cannot care for herself, has bad hygiene, Do not use for supports - use support_diet/hygiene instead |
| 5.0 | Poverty | Describes issues related to having low income or “money problems” | Use any time a respondent describes experiences/problems associated with lack of money or resources. Examples: having to work due to lack of money. Can be double-coded with stress codes if also identified as stressful. Do not use for financial support - instead use “support_financial” below. |
| 6.1 | Isolation | Describes issues associated with isolation. Could refer to either a cause or consequence of other problems as well. | Use with issues related to isolation, lack of social interaction, lack of support from family, community, etc. Examples would include “no support from family” or “being chased out of school”. Do not use for issues related to neglect by partner; instead use “domestic problems_neglect” for that. |
|  | Support | General support category used for sources or strategies of support. | Only use if no child categories are appropriate. Do not use for formal treatment approaches. |
| 17.0 | Support_Financial | Use with issues related to helping mother with financial support (giving her money) Can come from any source? | Eg: village saving & loan groups, help from family, etc. |
|  | Support_Religion | Describes the support through religion / spirituality and prayer | Use code in reference to religion as a support system (eg: church, church leader, prayer) |
|  | Support_Diet/Hygiene | Use with issues related to advice or help given on sanitation and cleanliness | eg: help her maintain her compound, clear the bush. Help prepare meals. |
|  | Support_Counsel | Describes advice from any informal source, distinct from mental health counseling | Informal or formal advice given by family, community, |
|  | Support_self | describes physical, social, or emotional acts the mother can take to help herself | Use to code source of support; can be double coded with type of support |
|  | Support_partner | describes physical, social, or emotional support from a partner | Use to code source of support; can be double coded with type of support |
|  | Support_peer | describes physical, social, or emotional support from a peer | Use to code source of support; can be double coded with type of support |
|  | Support_family | describes physical, social, or emotional support from a family | Use to code source of support; can be double coded with type of support |
|  | Support_community | describes physical, social, or emotional support from a community | Use to code source of support; can be double coded with type of support |
|  | Support_Transportation | Describes support related to transportation, getting to-from appointments, etc. | Use with issues related to escorting or transporting mother to facility for care/ treatment |
|  | Support_childcare | Describes support related to childcare | use for support from any source related to help with childcare. Do not use if support is specifically financial rather than behavioral (eg: helping care for children would be here - give money would be there) |
|  | Support_social | use to describe supports that are not tangible but more emotional in nature. | Examples: accept the mother, sitting together |
|  | Services/Treatment | Parent category to describe treatment strategies/sources. | Only use if not specific to below category. Distinct from clinic visits - those are the reasons for clinic visits, these are more strategies for intervention |
| 18.0 | Services_Facility/Hospital | describes any services delivered through a facility, clinic, hospital | can be double-coded with type of service |
|  | Services_Linkages | use for referrals to other medical services, police services, etc. | Ex: if the woman has severe injuries, we can send her to the hospital. |
|  | Services_Mobile | Describes any services delivered through mobile health teams, community health workers | Examples: Village health team (VHT), mobile clinic, home visit |
|  | Services_access | describes lack of or delayed treatment | Examples: delayed treatment, no treatment, lack early treatment, can’t get to clinic |
|  | Services_quality | Describes inadequate treatment | examples: no diagnosis, misdiagnosis, inadequate follow-up |
|  | Services_case management | used to describe actions associated with attending to psychosocial needs of patients, taking patient histories, following up on their service plan, etc. | Examples: case management, take patient history, patient monitoring, follow-up visits  Do not use for medication management - use services_medication for htat |
|  | Services_medication | Pharmaceuticals used as a form of treatment | Use for issues specific to ensuring adherence to medication for things like HIV, depression, epilepsy. For example, clinic visits for medication management. Eg: “do not be represented” means the patient should herself go to medication management appointments rather than have someone else go pick up her medication. |
|  | Services_counseling | Describes mental health counseling, couples counseling, etc. provided by a health clinic or other formal health care provider (VHT, etc.) | Use code for advice given from a source in the medical system such as health care provider. Do not use for advice/council from informational sources, community leaders, etc. Instead use support_council for that. |
|  | Services_pregnancy/family care | Describes family planning, perinatal care | Do not use if reason for clinic visit. Instead use clinic visit_family planning for that. This would be more an approach to intervention. |
|  | Services_health education | Describes services related to educating woman, family, community regarding health practices, available health services, etc. | Eg: mother needs to be educated about malaria, give nets, etc. |
